# Molecular landscapes of glioblastoma cell lines revealed a group of patients that do not benefit from *WWOX* tumor suppressor expression

**DOI:** 10.3389/fnins.2023.1260409

**Published:** 2023-09-15

**Authors:** Żaneta Kałuzińska-Kołat, Damian Kołat, Katarzyna Kośla, Elżbieta Płuciennik, Andrzej K. Bednarek

**Affiliations:** ^1^Department of Molecular Carcinogenesis, Medical University of Lodz, Lodz, Poland; ^2^Department of Functional Genomics, Medical University of Lodz, Lodz, Poland

**Keywords:** WWOX, glioblastoma, cell lines, patients, prognosis, molecular landscape, *in vitro*, bioinformatics

## Abstract

**Introduction:**

Glioblastoma (GBM) is notorious for its clinical and molecular heterogeneity, contributing to therapeutic failure and a grim prognosis. *WWOX* is one of the tumor suppressor genes important in nervous tissue or related pathologies, which was scarcely investigated in GBM for reliable associations with prognosis or disease progression despite known alterations. Recently, we observed a phenotypic heterogeneity between GBM cell lines (U87MG, T98G, U251MG, DBTRG-05MG), among which the anti-GBM activity of *WWOX* was generally corresponding, but colony growth and formation were inconsistent in DBTRG-05MG. This prompted us to investigate the molecular landscapes of these cell lines, intending to translate them into the clinical context.

**Methods:**

U87MG/T98G/U251MG/DBTRG-05MG were subjected to high-throughput sequencing, and obtained data were explored via weighted gene co-expression network analysis, differential expression analysis, functional annotation, and network building. Following the identification of the most relevant DBTRG-distinguishing driver genes, data from GBM patients were employed for, e.g., differential expression analysis, survival analysis, and principal component analysis.

**Results:**

Although most driver genes were unique for each cell line, some were inversely regulated in DBTRG-05MG. Alongside driver genes, the differentially-expressed genes were used to build a WWOX-related network depicting protein–protein interactions in U87MG/T98G/U251MG/DBTRG-05MG. This network revealed processes distinctly regulated in DBTRG-05MG, e.g., microglia proliferation or neurofibrillary tangle assembly. *POLE4* and *HSF2BP* were selected as DBTRG-discriminating driver genes based on the gene significance, module membership, and fold-change. Alongside *WWOX*, *POLE4* and *HSF2BP* expression was used to stratify patients into cell lines-resembling groups that differed in, e.g., prognosis and treatment response. Some differences from a WWOX-related network were certified in patients, revealing genes that clarify clinical outcomes. Presumably, *WWOX* overexpression in DBTRG-05MG resulted in expression profile change resembling that of patients with inferior prognosis and drug response. Among these patients, WWOX may be inaccessible for its partners and does not manifest its anti-cancer activity, which was proposed in the literature but not regarding glioblastoma or concerning *POLE4* and *HSF2BP*.

**Conclusion:**

Cell lines data enabled the identification of patients among which, despite high expression of *WWOX* tumor suppressor, no advantageous outcomes were noted due to the cancer-promoting profile ensured by other genes.

## 1. Introduction

Glioblastoma (GBM) is one of the most aggressive and frequently occurring brain tumors. Despite extensive research efforts and advancements in treatment modalities, the prognosis for GBM patients remains dismal, with a median survival of only around 12 months (Blakstad et al., 2023). The molecular complexity of glioblastoma entails significant challenges in understanding its mechanisms and developing effective therapeutic strategies (Singh et al., 2004; Pang et al., 2019). Combined with virtually inevitable relapse that only worsens the prognosis, the significance of subsequent research on this tumor entity is justified (Birzu et al., 2020; Oronsky et al., 2020). Pathogenetic GBM-related mechanisms are still far from being unraveled, and the application of current anti-glioblastoma therapies brings only a marginal improvement of the patients’ outcome due to the tumor location or acquired drug resistance (Stupp et al., 2009; Peignan et al., 2011; Delgado-Martin and Medina, 2020). One of the GBM traits hindering the effective treatment is the high heterogeneity that manifests on both intra-tumoral and inter-tumoral levels (Neftel et al., 2019; Dymova et al., 2021). Other clinical implications include the inconsistency between biomarkers identified in a single tumor biopsy or the need to consider clinically relevant subpopulations in planning of appropriate treatment regimen (Parker et al., 2015). This emphasizes a need for research identifying distinct molecular landscapes that may allow for a broader understanding of GBM complexity.

Recently, we investigated the phenotypic heterogeneity of four GBM cell lines (U87MG, T98G, U251MG, and DBTRG-05MG) and revealed some discrepancies in the results from *in vitro* assays following the ectopic overexpression of WW domain-containing oxidoreductase (*WWOX*) (Kaluzinska-Kolat et al., 2023; Varricchio et al., 2023). The literature on the role of this gene in brain development and pathology is generally of a reasonable extent and emphasizes its importance in nervous tissue (Kosla et al., 2020). However, it is acknowledged that less attention has been paid to determining the influence of *WWOX* on nervous system tumors than in other cancers (Winardi et al., 2013), even though *WWOX* affects the prognosis and treatment response of glioma (Liu et al., 2015). In GBM, the downregulation of this haploinsufficient tumor suppressor gene stems from the loss of heterozygosity and promoter methylation (Kosla et al., 2011). It has been shown that *WWOX* mitigates the glioblastoma’s infiltrative potential, and its loss can promote the migration of cancer cells (Chiang et al., 2012, 2013). Our previous research concluded that the anti-GBM activity of *WWOX* is mainly a consequence of reduced cell viability and invasion (Kaluzinska-Kolat et al., 2023). However, while most of the data indicated the antineoplastic activity of *WWOX*, discrepancies related to colony growth and formation were noticed in one of the above GBM cell lines, i.e., DBTRG-05MG. This suggests that DBTRG-05MG exhibited a distinct expression profile in comparison to U87MG/T98G/U251MG, which prompted us to perform a High-throughput sequencing (HTS) on cellular variants from the previous study, with an intention to identify the differences that may help in stratifying GBM patients into groups of various clinical outcomes. The usage of high-throughput sequencing technologies over the years has played a considerable role in elucidating the molecular intricacies of glioblastoma (Jovcevska, 2020). To ensure the robustness of data in such studies, it is advisable to prepare biological replicates for RNA-sequencing (RNA-Seq), seeing that the use of only technical replicates is uncertain (Blainey et al., 2014). The current study considered this recommendation and aimed to identify molecular landscapes that distinguish DBTRG-05MG from U87MG/T98G/U251MG and may clarify the phenotypic *WWOX*-related differences or translate them into the clinical context.

## 2. Materials and methods

### 2.1. Cell culture, stable transduction, and confirmation of acquiring cellular variants

The cell culture, procedure of lentiviral transduction for *WWOX* upregulation, and its confirmation are described in our preceding study (Kaluzinska-Kolat et al., 2023). Briefly, four cell lines representing glioblastoma (U87MG, T98G, U251MG, DBTRG-05MG) were purchased from the European collection of cell cultures (ECACC), incubated at 37°C in a humidified atmosphere of 5% CO_2_, and cultured in MEM (T98G), EMEM (U87MG and U251MG) or RPMI-1640 (DBTRG-05MG) medium supplemented with L-glutamine, fetal bovine serum, and Antibiotic-Antimycotic. To overexpress *WWOX*, stable transduction was performed via the GIPZ™ lentiviral system in a polybrene-containing starvation medium. Following the exchange of viral medium to full medium, the clonal selection was performed using puromycin. In each glioblastoma cell line, the stable transductants represented either the “WWOX” cellular variant (treated with the GIPZ™ system) or the “Vec” cellular variant (treated with the control Puro-Blank system). After protein extraction, the efficiency of transduction was confirmed by Western Blot analysis. Additional characterization of four cell lines is summarized in Supplementary Table S1; data included the genetic alterations acquired from Cancer Cell Line Encyclopedia (Ghandi et al., 2019), as well as the putative disease subtypes obtained from the literature (Zhong et al., 2014; Kiseleva et al., 2016; Salvadores et al., 2020; Oliveira et al., 2022; Qiu et al., 2023; Varricchio et al., 2023).

### 2.2. Isolation of RNA, preparation of CAGE library, sequencing, mapping, and counting tags

Total RNA was isolated using an Extracol reagent per the manufacturer’s protocol (EURX, Gdansk, Poland). The quality of total RNA was assessed by Agilent 2,100 Bioanalyzer (Agilent Technologies, Santa Clara, CA, United States) to confirm that RNA integrity number is over 8.0. Reverse transcription of RNA was performed using random primers (CAGE library preparation Kit; K.K. DNAFORM, Yokohama, Japan). The selection of RNA/cDNA hybrids was enabled by cap-trapping on streptavidin beads. Following the digestion of RNA using RNaseI/H, the linker ligation to 5′ and 3’ cDNA ends enabled the construction of double-stranded cDNA libraries. Sequencing of libraries using the Cap analysis gene expression (CAGE) method was performed using 75 nt single-end reads on a NextSeq 2000 instrument (Illumina, San Diego, CA, United States). Data from this experiment are deposited in Gene expression omnibus (GEO) database (accession number GSE229210). The quality of the obtained data was evaluated via the FastQC tool (v0.11.9). Reads alignment and mapping to a human reference genome (hg38) were performed using the STAR method (Dobin et al., 2013). Counting tags at each side was performed using SAMtools in R (Rsamtools v2.2.3). In each cell line, CAGE-Seq was performed in biological triplicate for the “WWOX” and “Vec” cellular variants.

### 2.3. Bootstrapping and transforming CAGE-seq data, weighted gene co-expression network analysis (WGCNA), identification and functional annotation of driver genes

Read counts were subjected to the resampling bootstrap method (Al Seesi et al., 2014; Kulesa et al., 2015) to acquire a relevant quantity of samples for Weighted gene co-expression network analysis (WGCNA) (Qu et al., 2021). The latter was initially performed to obtain the consensus modules across cell lines (temporarily excluding DBTRG-05MG due to technical issues preventing analysis) via Biological network reconstruction omnibus (BioNERO) (Almeida-Silva and Venancio, 2022) and further using the classical WGCNA approach (Langfelder and Horvath, 2008) to investigate all four cell lines independently. At the BioNERO stage, bootstrapped counts were subjected to variance stabilizing transformation (Love et al., 2014) via the vstransform parameter set to “TRUE” and filtered by variance using variance_filter set to “TRUE” with n = 10,000 during the automatic data preprocessing. Consensus modules identified during BioNERO workflow (via consensus_modules() and consensus_trait_cor() functions) were further investigated using the classical WGCNA approach. The transformation into an adjacency matrix was performed with power = 9 selected in virtue of the pickSoftThreshold() function with signed hybrid network type and with the scale-free topology fitting index (R2) > 0.8. Following the topological overlap matrix preparation, the hierarchical clustering was performed through the hclust() function with an average agglomeration method. Established genes-containing modules were correlated to a binary trait representing stable transductants (samples were denoted as “WWOX” or “Vec” according to the procedure they were subjected to during stable transduction). Expression profiles were visualized using a heatmap.2() function of gplots v3.1.1 package with Euclidean distance metric for row ordering. Identification of genes most correlated with both module and trait (henceforth referred to as “driver genes” or “drivers”) was facilitated via the verboseScatterplot() function with threshold ≥0.7 for both Gene significance (GS) and Module membership (MM). Expression of driver genes was also visualized using gplots v3.1.1 package. Subsequently, the intramodular connectivity of top genes was established via exportNetworkToVisANT() function of WGCNA with further manual adjustment using VisANT 5.0 (Granger et al., 2016) and Cytoscape 3.9.0 (Shannon et al., 2003). The top 50 genes within a module for each cell line were obtained except DBTRG-05MG, for which the top 25 genes were acquired from the module part consistent with other cell lines and the top 25 from the module part with the unique expression profile. Subsequently, driver genes for each cell line were independently subjected to the Gene-set enrichment analysis (GSEA) using the R-package WebGestaltR (Liao et al., 2019) with a functional annotation database set to “gene ontology (GO) – biological process.” The top 20 annotations were retrieved for each list of genes (i.e., drivers combined from two modules of interest described in section 3.1). A minimum number of three query genes for a specific annotation was set. Genes were ranked on the basis of their log_2_ fold-change (log_2_FC) values acquired from the limma-voom, which is described in the subsequent section. Lastly, an intersection analysis between lists of driver genes for all cell lines was performed using the UpSetR package in the R environment (Conway et al., 2017); for this step, the threshold of GS and MM was set to ≥0.65.

### 2.4. Differential expression analysis (cell lines data), building and annotating the network

Bootstrapped counts were also subjected to Differential expression analysis (DEA) using the limma-voom method (Law et al., 2014; Ritchie et al., 2015). Preprocessing data via calcNormFactors() and filtering lowly expressed genes (requirement of minimum ≥5 counts per million in ≥1 library) were followed by transformation via the voom() function. The model was fitted in limma using weighted least squares for each gene via lmFit(), and log_2_FC values for “WWOX” (case) versus “Vec” (control) in each cell line were acquired following the execution of makeContrasts() function with default parameters. Empirical Bayesian smoothing of standard errors preceded the acquisition of the top-ranked genes via the topTable() function. At this stage, it was intended to compare differences between cell lines and not only between cellular variants within a specific cell line. Thus, the typically reasonable log_2_FC threshold of |0.57| was reduced to |0.37| to facilitate investigating the distinct expression profile of DBTRG-05MG versus the other three cell lines. In the middle of the study workflow, this approach was considered justifiable since it allowed to identify inversely regulated genes whose expression was, e.g., gently increased in the “WWOX” variant compared to the “Vec” DBTRG-05MG, but at the same time evidently downregulated in other cell lines. Genes identified through this approach served as an input for Cytoscape-integrated NDEx iQuery (Pillich et al., 2023) that enabled the construction of a protein–protein interaction network using data from the BioGRID repository (Oughtred et al., 2021). Moreover, the input list contained the driver genes simultaneously identified in at least three cell lines, as well as drivers that exhibited inverse properties in DBTRG-05MG versus U87MG/T98G/U251MG. These genes were identified using methods described in the previous section. If drivers presenting inverse regulation in DBTRG-05MG were not a part of an automatically built network, they were added manually via the Advanced network analysis tool (ANAT) (Signorini et al., 2021). Afterwards, the “STRINGify network” option of Cytoscape’s plugin “stringApp” (Doncheva et al., 2019) was run onto the entire network to expand it with additional proteins that demonstrated a very high interaction score (confidence of ≥0.9). After the EntOpt Layout was applied to maximize the clarity of the network, log_2_FC values for all cell lines were imported from a separate text file and visualized inside nodes via the Cytoscape’s plugin “enhancedGraphics” (Morris et al., 2014). Finally, network was subjected to the Over-representation analysis (ORA) via WebGestaltR with top 20 annotations retrieved for each list of genes (see section 3.3 for details) and minimum number of three query genes for a specific annotation.

### 2.5. Patients’ data acquisition, designating groups, and survival analysis

The remaining methodological parts were intended to certify the cell lines-related observations using data from glioblastoma patients. RNA-Seq quantification files generated using STAR workflow (Dobin et al., 2013) were acquired from Genomic Data Commons (GDC) for glioblastoma patients of The Cancer Genome Atlas (TCGA-GBM) using the GDCRNATools v1.18.0 package in the R environment v4.2.3. Together with *WWOX*, the two most important driver genes discerning DBTRG-05MG from other cell lines (i.e., *POLE4* and *HSF2BP*; see section 3.4 for details) were considered a geneset of which expression signature could reflect the expression profile change that occurred in cells following stable lentiviral transduction. Independent groups of patients representing (1) “Vec” of U87MG/T98G/U251MG, (2) “WWOX” of U87MG/T98G/U251MG, (3) “Vec” of DBTRG-05MG, and (4) “WWOX” of DBTRG-05MG were determined on the basis of *WWOX*/*POLE4*/*HSF2BP* expression denoted as “high” (↑) or “low” (↓); specific cut-off values summarized in Supplementary Table S2. Together with designating cellular variants-resembling groups, the acquisition of corresponding clinical data for TCGA-GBM patients from GDC enabled the analysis of Overall survival (OS) and Disease-free survival (DFS). Prior to focusing on designated groups, TCGA-GBM patients were subjected to DFS analysis via Evaluate Cutpoints tool (Ogluszka et al., 2019) based on RNA-Seq expression data separately for *WWOX*, *POLE4*, and *HSF2BP*. Subsequently, patients with desired *WWOX*/*POLE4*/*HSF2BP* expression were selected via which() function in the R environment and further subjected to survival analysis using the surfvit(), of which results were visualized using ggsurvplot(). For each patient from the designated groups, the supplementary clinical information and data on molecular subtypes were acquired from the literature (Brennan et al., 2013; Wang et al., 2017; Zakharova et al., 2022), whereas the drug sensitivity prediction for Temozolomide (TMZ) measured by Half-maximal inhibitory concentration (IC50) was obtained from the CancerRxTissue repository (Li et al., 2021). These additional data were visualized using pROC and ggplot2 R-packages (Robin et al., 2011).

### 2.6. Differential expression analysis (patients’ data) and principal component analysis

After the groups of patients were established, they were subjected to DEA performed via limma-voom as in section 2.5, except for the log_2_FC threshold set to |0.57| at the current stage. Two comparisons were considered. To resemble the *WWOX*/*POLE4*/*HSF2BP* expression changes that were corresponding in U87MG/T98G/U251MG cell lines, patients representing the “WWOX” cellular variant (*WWOX*↑ *POLE4*↓ *HSF2BP*↓) were compared to those representing the “Vec” variant (*WWOX*↓ *POLE4*↑ *HSF2BP*↑). In contrast, reflecting the expression changes in the “WWOX” variant versus the “Vec” variant of DBTRG-05MG required patients with *WWOX*↑ *POLE4*↑ *HSF2BP*↑ or *WWOX*↓ *POLE4*↓ *HSF2BP*↓ profile, respectively. Expression of genes regulated inversely between two comparisons was visualized on heatmap using gplots v3.1.1 as mentioned in section 2.3; the same genes were also functionally annotated using ORA as in section 2.4. At the same stage, subsidiary analysis of immune infiltration and gene expression implicated in the myeloid-related transcriptional states was performed among patients subjected to DFS analysis. Infiltration estimates were obtained via TIMER 2.0 (Li et al., 2020); the expression profile of patients was uploaded to the “Estimation” module of TIMER 2.0 with the “GBM” option under the “cancer type.” The list of genes implicated in the myeloid-related transcriptional states was acquired from the literature (Gangoso et al., 2021; Ravi et al., 2022; Rajendran et al., 2023). Gene expression and infiltration estimates were visualized on the violin plots using the ggpubr v0.6.0 package in R environment v4.2.3. Afterwards, the results of patients-related DEA were explored for expression changes that reflect the fold-change data from cell lines implemented in the interaction network from section 2.4. Expression of identified genes was used for dimensional grouping performed via the Principal component analysis (PCA). These genes were treated as active variables, of which contribution to spatial partitioning across the first two principal components was visualized via FactoMineR and factoextra R-packages (Lê et al., 2008). The biplot of both quantitative and qualitative variables was visualized using the fviz_pca_biplot() function with a confidence ellipse drawn for each group of patients. Additionally, gene expression differences between groups of patients were investigated for statistical significance and visualized as violin plots using the vioplot R-package.

### 2.7. Statistical analysis

GraphPad Prism v8 (GraphPad Software, San Diego, CA, United States) was employed for statistical analysis. The normality of distribution was determined by the Shapiro–Wilk test. The unpaired t-test or the Mann–Whitney test was used depending on the data distribution. Results with a value of p less than 0.05 were considered statistically significant.

## 3. Results and discussion

### 3.1. Gene modules that were consistent in U87MG, T98G, and U251MG, were not entirely coherent in DBTRG-05MG

**Figure 1:**
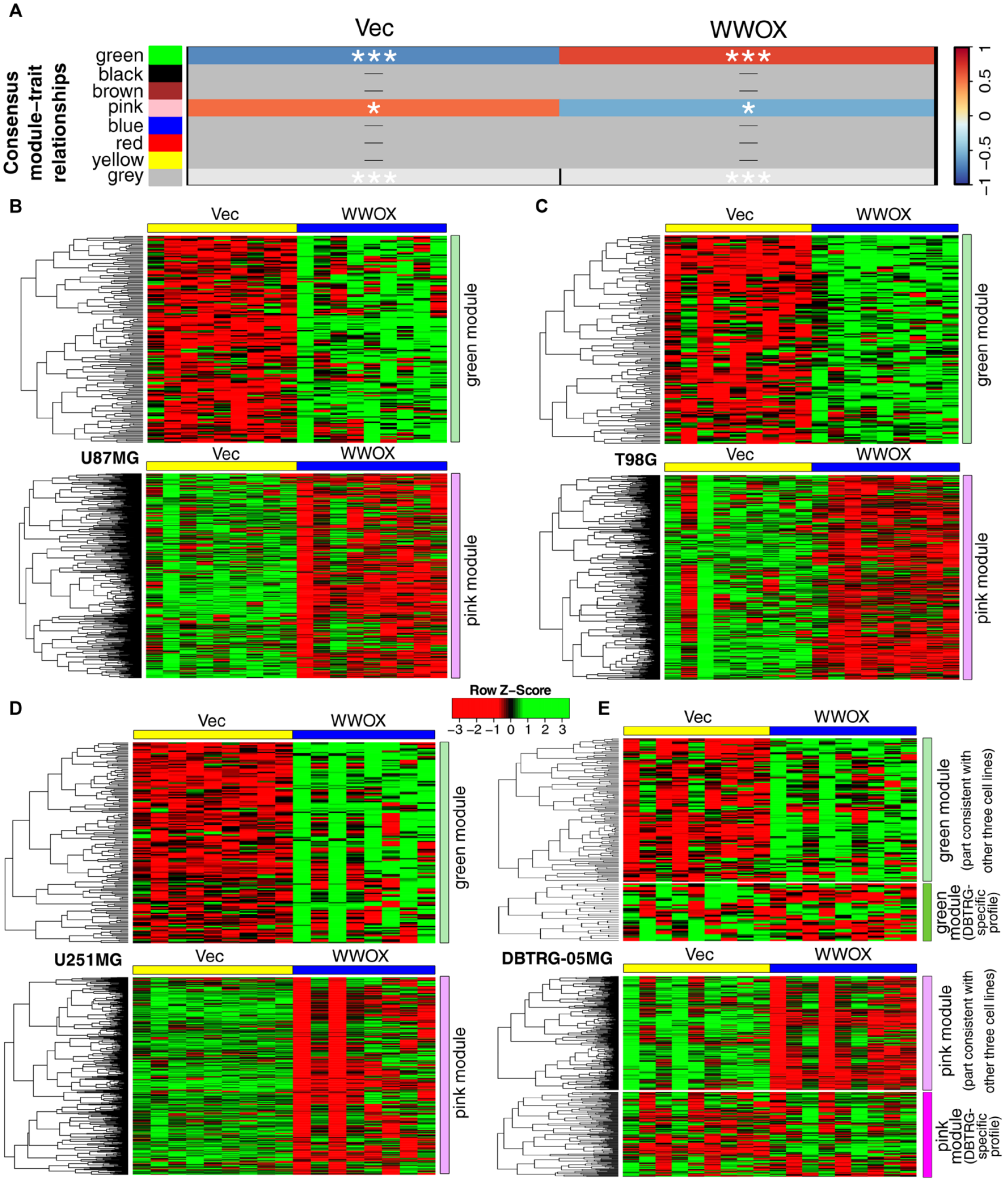
Expression profile of green and pink consensus modules in all cell lines included in the study. **(A)** The consensus module-trait relationship calculated via BioNERO/WGCNA indicated that green and pink consensus modules were congruous in **(B)** U87MG, **(C)** T98G, or **(D)** U251MG. In contrast, the same modules were not entirely coherent in **(E)** DBTRG-05MG, for which an inverse profile was noticed in a portion of each consensus module. *p* < 0.05 (*), *p* < 0.001 (***).

The initial step of the current analysis was intended to identify a common expression profile for U87MG, T98G, and U251MG cell lines owing to their coherence in results from the previous study following *WWOX* overexpression (Kaluzinska-Kolat et al., 2023). DBTRG-05MG was temporarily excluded from establishing consensus module-trait relationships since it caused technical issues preventing analysis. Nonetheless, the input gene list to this step was determined after CAGE-Seq data filtering to retain genes whose expression varied between “Vec” and “WWOX” samples for each cell line independently, including DBTRG-05MG. Up to 10,000 most variable genes per cell line were acquired, with 5,744 variable genes present in all four lists of genes (Supplementary Table S3). This geneset served as the input to the BioNERO pipeline that revealed two consensus modules, i.e., green (159 genes) and pink (525 genes), that distinguish “Vec” and “WWOX” cellular variants of U87MG, T98G, and U251MG cell lines (Figure 1A). These modules were visualized separately for each cell line (Figures 1B–D), including DBTRG-05MG (Figure 1E), of which the expression profile was not entirely consistent with U87MG, T98G, and U251MG. Approximately 20% of the green module (32 genes) and about 30% of the pink module (172 genes) tended to be inversely regulated in DBTRG-05MG compared to other cell lines. The distinct expression profile of DBTRG-05MG might coincide with the gene mutation profile that is much more altered in this cell line than in others. According to the Catalogue of somatic mutations in cancer (COSMIC), mutation quantity is doubled in DBTRG-05MG relative to U251MG and U87MG or is 1.6 times higher than in T98G (Forbes et al., 2011).

### 3.2. Cell lines were characterized by unique driver genes, but some of the overlapping ones exhibited inverse properties in DBTRG-05MG

**Figure 2:**
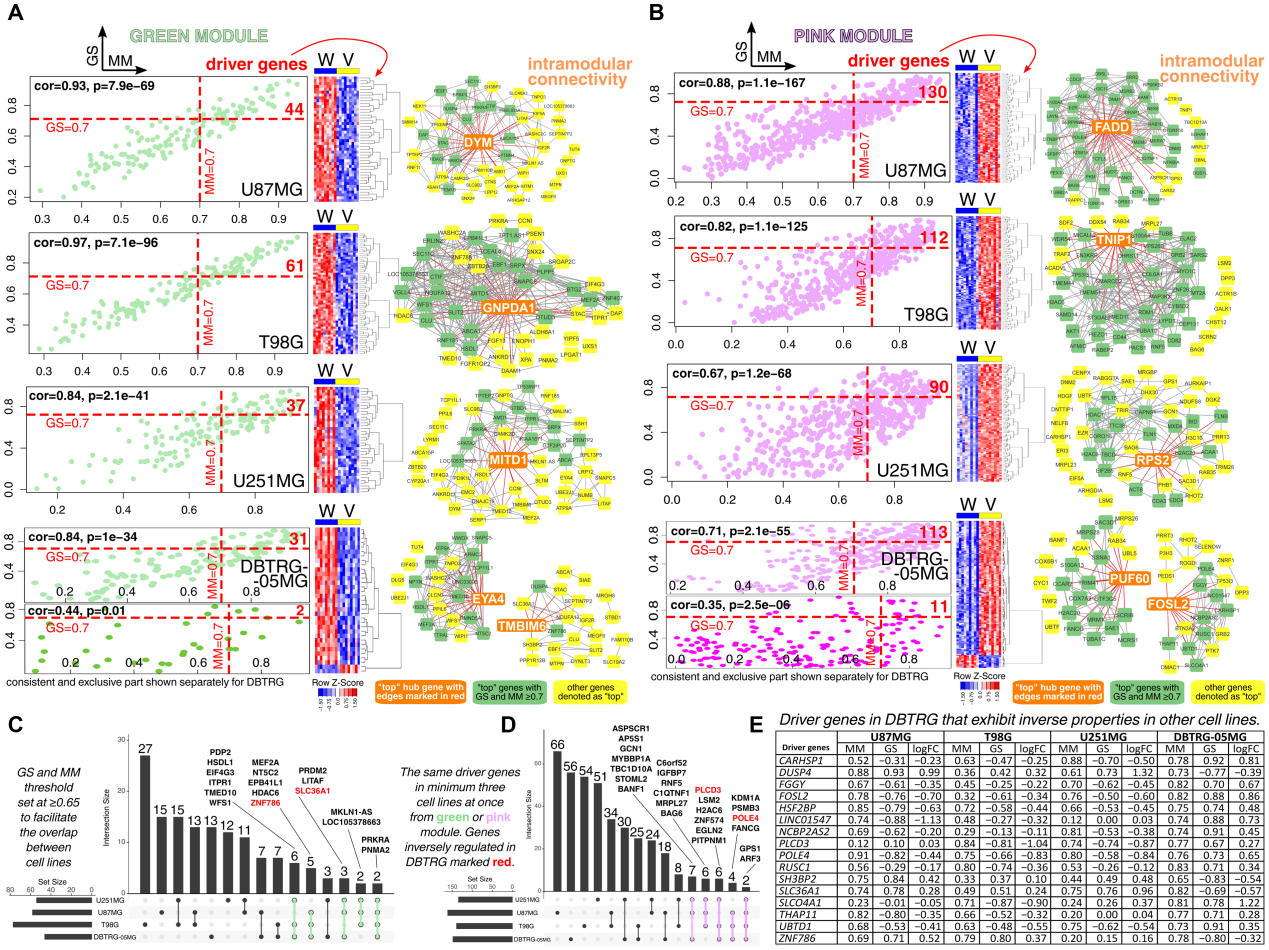
Investigation of driver genes for all cell lines with an emphasis on drivers that exhibit inverse properties in other cell lines relative to DBTRG-05MG. Separately for U87MG, T98G, U251MG, and DBTRG-05MG, the driver genes from **(A)** green module or **(B)** pink module were identified via WGCNA using GS and MM threshold of ≥0.7. The part of the green or pink module that was consistent in DBTRG-05MG and other cell lines was presented separately from the part with an inverse profile found exclusively in DBTRG-05MG. Intramodular connectivity for top genes was additionally included. Overlap of driver genes between cell lines was presented separately for **(C)** green and **(D)** pink modules. **(E)** Some driver genes in DBTRG-05MG were characterized by inverse GS and log_2_FC relative to other cell lines. For example, *POLE4* was identified in the pink module, and the expression of this gene is higher in “Vec” variants of U87MG, T98G, and U251MG. In other words, *POLE4* is negatively correlated to “WWOX” variants of these cell lines since the negative GS and log_2_FC are present. Regarding DBTRG-05MG, *POLE4* expression was higher in the “WWOX” variant relative to “Vec”; thus, positive GS and log_2_FC values are visible.

Insights into the WGCNA revealed that there are dozens to over a hundred cell line-specific driver genes, i.e., genes most correlated with both module and trait (Figures 2A,B). Herein, the threshold was set to ≥0.7 for both GS and MM. Some DBTRG-specific drivers met these requirements within unique parts of two gene modules for this cell line (*DUSP4* and *ZNF786* for green module; *CARHSP1*, *FGGY*, *FOSL2*, *HSF2BP*, *LINC01547*, *NCBP2AS2*, *POLE4*, *RUSC1*, *SLCO4A1*, *THAP11*, and *UBTD1* for pink module). A short synopsis for those having the literature data in the context of GBM will be provided at the end of this section. Complete lists of driver genes and related GSEA are summarized in Supplementary Table S4. In brief, functional annotation revealed that T98G-specific drivers regulate dendrite development or actin filaments, while U87MG-specific drivers control development growth or vesicle-mediated transport in the synapse. Moreover, U251MG-specific drivers alter cell polarity or cellular drug response, whereas DBTRG-specific drivers orchestrate proliferation or axon development. Investigating intramodular connectivity of green or pink module for each cell line indicated some genes being top hubs, i.e., *DYM*, *FADD*, *GNPDA1*, *TNIP1*, *MITD1*, *RPS2*, *EYA4*, *TMBIM6*, *PUF60*, and *FOSL2* (all were driver genes except for *TMBIM6* that presented MM ≥ 0.7 but GS < 0.7). For some of these genes, their role in GBM is documented. Overexpression of Fas-associated *via* death domain (*FADD*) may sensitize GBM to cell death (Marin-Rubio et al., 2019). TNFAIP3-interacting protein 1 (*TNIP1*) mediates a signaling cascade that sustains GBM proliferation; a high level of *TNIP1* was correlated with poor survival (Lei et al., 2020). Elevated expression of Microtubule interacting and trafficking domain-containing 1 (*MITD1*) forecasted unfavorable prognosis in patients with low-grade glioma and GBM. For the latter, *MITD1* was positively correlated to homologous recombination deficiency (Dong et al., 2022). The gene Eyes absent homolog 4 (*EYA4*) plays a putative tumor-promoting role in nervous system tumors (Chong et al., 2023) and was found to promote cell proliferation in GBM (Li et al., 2018b). Transmembrane BAX inhibitor motif containing 6 (*TMBIM6*) is elevated in GBM but may be downregulated in other tumor entities; it may be associated with tumor growth and dependent signaling (Yi et al., 2023). Knockdown of the Poly(U) binding splicing factor 60 (*PUF60*) reduced GBM tumorigenicity and proliferation (Wang et al., 2022).

Subsequently, intersection analysis of the driver genes for all cell lines was performed to assess whether there are common genes for U87MG, T98G, U251MG, and DBTRG-05MG. At this stage, GS and MM threshold was set at ≥0.65 to facilitate the overlap. Four drivers (two in green and two in pink module) were found in all cell lines and presented congruent expression change between “WWOX” and “Vec” cellular variants; these were *PRKRA* and *PNMA2* (green module) or *GPS1* and *ARF3* (pink module). The high expression ratio of the Protein activator of interferon-induced protein kinase (*PRKRA*) and its presumably oncogenic antisense counterpart *CHROMR* was linked to favorable survival of GBM patients (Sirvinskas et al., 2023). PNMA family member 2 (*PNMA2*) was downregulated in pediatric GBM cell line SJ-GBM2 following irradiation and might positively regulate the apoptotic process (Alhajala et al., 2018). No precise data on the role of *GPS1* and *ARF3* in GBM were found, although the latter gene might potentiate G_1_/S cell cycle transition, as noted in breast cancer (Casalou et al., 2020).

Of driver genes found in at least three cell lines, only those of DBTRG-05MG were inversely regulated in comparison to U87MG, T98G, or U251MG (Figures 2C,D). This suggested that particular attention should be drawn to driver genes of DBTRG-05MG that exhibit inverse relation to the “WWOX” variant relative to “Vec,” as estimated by GS or MM, as well as log_2_FC from DEA performed in parallel (Figure 2E). Reducing the GS and MM threshold to ≥0.65 enabled the acquisition of three drivers (i.e., *PLCD3*, *SH3BP2*, and *SLC36A1*) in addition to those mentioned at the beginning of this section. Some of these drivers have documented function in glioblastoma. Dual-specificity phosphatase (DUSP) family regulates GBM sensitivity to treatment (Prabhakar et al., 2014); specifically for *DUSP4*, its overexpression in glioblastoma significantly reduced proliferation and colony formation (Waha et al., 2010). Knockdown of Calcium regulated heat stable protein 1 (*CARHSP1*) alleviated glioblastoma radioresistance, presumably via the inflammatory signaling pathway. When receiving radiotherapy, individuals with higher *CARHSP1* levels had unfavorable survival (Zhu et al., 2021). Fos-like 2 (*FOSL2*) regulates angiogenesis and plasticity of GBM, is involved in tumor development, and regulates the so-called natural evolution signature that contains genes most differentially expressed between new and old GBM lesions (Marques et al., 2021; Wan et al., 2021; Eisenbarth and Wang, 2023b). Furthermore, it was hailed as one of six master regulators of mesenchymal gene expression signature (Fedele et al., 2019). Phospholipase C delta 3 (*PLCD3*) was correlated to diacylglycerol and is converted to phosphatidic acids by diacylglycerol kinase α, of which inhibition was recently considered a promising strategy for GBM (Ohanian and Ohanian, 2001; Wang et al., 2021; Purow, 2022). Lastly, although Solute carrier family 36 member 1 (*SLC36A1*) expression presented high variability among GBM individuals, it was higher than in control samples (i.e., human cortex) (Schaffenrath et al., 2021). Interestingly, this can bring benefits to the clinic since *SLC36A1* has been utilized as a drug-delivery platform for therapeutics (Thwaites and Anderson, 2011).

### 3.3. WWOX, drivers, and related genes formed a network that was of high interconnectivity and was related to various biological processes, including those associated with the nervous system

**Figure 3:**
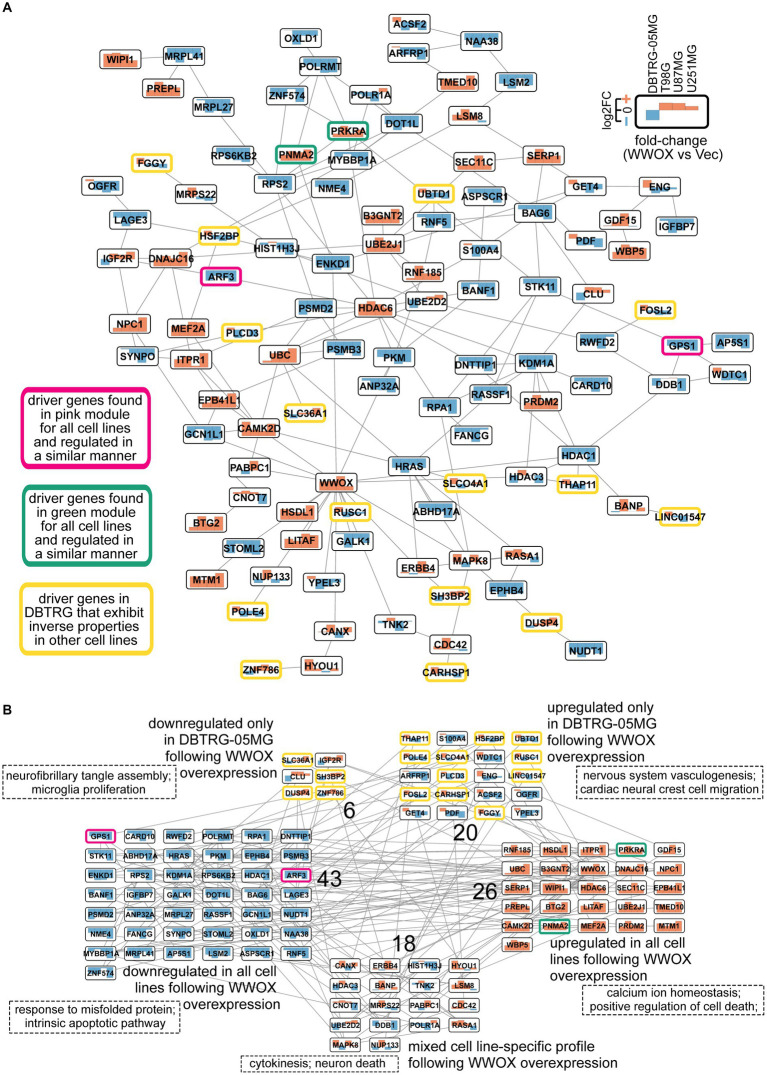
The WWOX-related network containing genes from DEA, drivers from WGCNA, and top interactors from STRING. **(A)** EntOpt Layout was adopted to maximize the clarity of the network. The log_2_FC values (“WWOX” versus “Vec” comparison) for all cell lines were included to facilitate the interpretation; see the top-right corner for the legend. **(B)** The same network was visualized using Grid Layout to group nodes according to their fold-change. Next to each group, a number representing the quantity of nodes is displayed. Groups were independently subjected to gene ontology analysis; two annotations per group are provided in dashed rectangles. The top 20 annotations per group are summarized in Supplementary Table S5.

Following WGCNA and DEA (described in sections 2.3 and 2.4, respectively), it was decided to build a *WWOX*-related network (Figure 3). Included genes were the drivers identified in at least three cell lines (GS and MM ≥0.65) but also the “background” genes from DEA (log_2_FC ≥ |0.37|) and from the STRING database (genes with a very high interaction score, i.e., the confidence of ≥0.9). Details on network construction are provided in section 2.4. Such an approach to elaborating the network has enabled the identification of other genes that were not considered drivers but still exhibited the inverse expression profile in DBTRG-05MG relative to U87MG, T98G, and U251MG. These genes were *ACSF2*, *ARFRP1*, *CLU*, *ENG*, *GET4*, *IGF2R*, *OGFR*, *PDF*, *S100A4*, *WDTC1*, and *YPEL3* (Figure 3A). Acyl-CoA synthetase family member 2 (*ACSF2*) may regulate neuronal development, and even though it did not affect anchorage-dependent growth in GBM cells, it is related to *ACSVL3* that supports glioblastoma stem cell maintenance and tumorigenicity (Sun et al., 2014; Tomi-Andrino et al., 2022). The association between ADP ribosylation factor-related protein 1 (*ARFRP1*) and a higher risk of GBM-type glioma requires further investigation (Song et al., 2012) since available data are inconsistent (Atkins et al., 2019; Ali et al., 2021). Clusterin (*CLU*) levels were found elevated in glioblastoma stem cells which induced an anti-apoptotic state (Osuka et al., 2021); however, it is worthy of note that *CLU* can also promote apoptosis when localized in the nucleus (Kim and Choi, 2011) (the first mentioned reference investigated a secreted protein form). Endoglin (*ENG*) is an angiogenic biomarker determining the microvessel density of GBM (Afshar Moghaddam et al., 2015). Although its prognostic significance is inconsistent, it is overexpressed in actively proliferating endothelial cells and supports new vascular networks (Bastos et al., 2022). Insulin-like growth factor 2 receptor (*IGF2R*) is overexpressed in GBM samples compared with normal brain specimens, but no association between its expression and patients’ outcomes was observed (Maris et al., 2015). The same authors underlined that the reports on the entire IGF family in glioblastoma are typically restricted to small series and have not yielded consistent results. Targeting the mitochondrial Peptide deformylase (*PDF*) was suggested as a promising approach for sensitization of GBM to chemotherapy; data were obtained from research that investigated an inhibitor of PDF, i.e., actinonin, that led to the activation of mitochondrial unfolded protein response, increased mitochondrial fission, as well as promoted the integrated stress response to increase apoptosis (Lan et al., 2022). S100 calcium-binding protein A4 (*S100A4*) was found to be necessary for maintaining glioblastoma stem cells self-renewal; moreover, ablation of *S100A4*-expressing cancer cells is sufficient to impede glioma growth *in vivo* (Chow et al., 2017).

From another point of view, the elaborated network can be grouped on the basis of fold-change values established between “WWOX” and “Vec” variants in U87MG, T98G, U251MG, or DBTRG-05MG (Figure 3B). Although most of the included genes had consistent profiles in all cell lines, the observations unique for DBTRG-05MG constituted more than 20% of the entire graph (26 genes out of 113). Functional annotation revealed that misfolded protein and cell death were similarly affected in all cell lines following *WWOX* overexpression. On the contrary, *WWOX* overexpression regulated microglia proliferation, neurofibrillary tangle assembly, and nervous system vasculogenesis differently in DBTRG-05MG than in other cell lines. A more detailed summary of gene ontology is collected in Supplementary Table 5. Some annotated processes can be certified using data from our previous research (Kaluzinska-Kolat et al., 2023). ORA of the entire network and the parts consistent for all cell lines has repeatedly indicated cell death and apoptotic signaling pathway regulation. Indeed, all four “WWOX” cellular variants were characterized by decreased cell viability, alongside the intensified apoptosis of T98G/DBTRG-05MG/U87MG cells following *WWOX* overexpression. Results for U251MG were statistically insignificant, although a consistent trend was visible (Kaluzinska-Kolat et al., 2023). Part of the network specific for DBTRG-05MG suggested that vascular tube formation might differ this cell line from the others; however, there was no *in vitro* assay investigating this process in our former study. Nevertheless, annotations indicating a unique regulation of proliferation align with previous data – proliferative potential after *WWOX* overexpression was increased only in DBTRG-05MG (Kaluzinska-Kolat et al., 2023). Annotated processes in each group of the network seem to be related; for example, the association between neural crest cells and vasculogenesis is known (Bergwerff et al., 1998; Wiszniak et al., 2015). Moreover, microglia cells are observed around Neurofibrillary tangles (NFTs), i.e., aggregates of hyperphosphorylated tau protein that are neuropathological indicators of Alzheimer’s disease (AD) (Metaxas and Kempf, 2016). Although microglia cells can ingest NFTs, they do so inefficiently and may release tau aggregates into the environment, propagating disease pathology (Hopp et al., 2018; Perea et al., 2018). The functionality of tau protein in brain pathologies is not only limited to AD; its role in GBM is also documented (Pagano et al., 2021; Hedna et al., 2022).

### 3.4. Stratification by expression of *WWOX* and the two most relevant drivers explaining DBTRG-05MG otherness (*POLE4* and *HSF2BP*) showed that patients differ in survival

**Figure 4:**
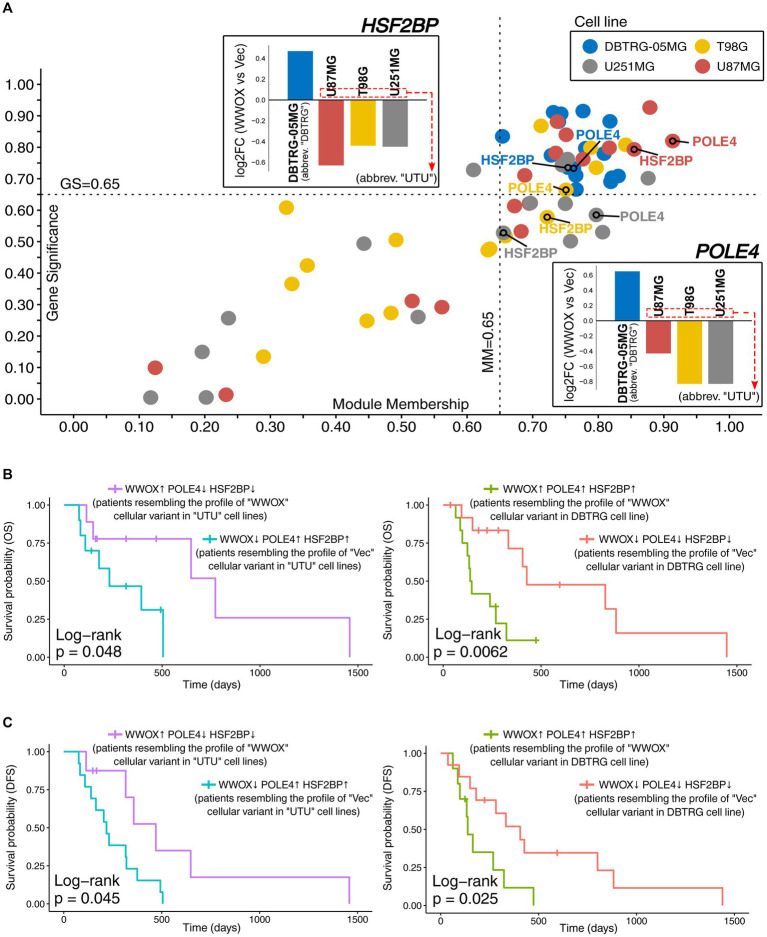
Justifying the selection of *POLE4* and *HSF2BP* as the two most relevant drivers explaining the otherness of DBTRG-05MG. **(A)** Scatterplot presenting GS and MM of all DBTRG-discerning driver genes that were summarized in Figure 2E. *POLE4* and *HSF2BP* are annotated to emphasize the relevance of their GS and MM values accumulating in the top-right corner for all cell lines. For this step, absolute GS values were used to facilitate the visualization of data from all cell lines at once. The log_2_FC values are also provided to illustrate the dissimilarity between the “WWOX” versus “Vec” comparison in DBTRG-05MG. Alongside *WWOX*, the expression of *POLE4* and *HSF2BP* was used to stratify GBM patients in groups resembling cellular variants of U87MG, T98G, and U251MG cell lines (abbreviated as “UTU” from the first letters of their names), as well as separately the variants of DBTRG-05MG (abbreviated as “DBTRG”). Data for patients presenting the differences in **(B)** OS and **(C)** DFS are summarized in Supplementary Table S2.

Analysis was directed toward verifying data from cell lines using GBM patients. However, there was a need to reflect the dissimilarity observed in DBTRG-05MG relative to U87MG, T98G, and U251MG, the last three abbreviated as “UTU” from the first letters of cell line names. Thus, the two most relevant driver genes of DBTRG-05MG discerning this cell line from others were selected alongside *WWOX* to stratify patients and elaborate the groups resembling “Vec” and “WWOX” cellular variants. The most promising drivers from Figure 2E appeared to be *POLE4* and *HSF2BP*, of which GS, MM, and log_2_FC values distinguished DBTRG-05MG from other cell lines. GS and MM values for all DBTRG-discerning drivers were visualized on a scatterplot emphasizing two selected genes; inverse fold-changes between DBTRG-05MG versus others were included (Figure 4A). After designating groups (see section 2.5 for details), patients resembling “WWOX” variants of “UTU” cell lines were compared to those representing “Vec” variants in the same cell lines. Likewise, individuals similar to the “WWOX” variant of DBTRG-05MG were compared to patients showing a “Vec”-like profile in this cell line. Only ~30% of patients from the TCGA-GBM cohort had an adequate expression profile of *WWOX*, *POLE4,* and *HSF2BP*. Survival analysis revealed that patients resembling the profile of the “WWOX” cellular variant in “UTU” cell lines had longer OS and DFS than those resembling the “Vec” variant. On the other hand, contrary results were observed for individuals representing the cellular variants of DBTRG-05MG. Overall, *WWOX* appears to be less significant in the presence of *POLE4* and *HSF2BP*, although subsequent studies on larger datasets are required to evaluate their relation thoroughly. When analyzing each gene individually, DFS data from the TCGA-GBM cohort indicate a favorable effect of higher *WWOX* expression, whereas higher *POLE4* or *HSF2BP* expression is somewhat unfavorable (Supplementary Figure S1). In further steps, we focused on patients representing DFS outcomes since the events caused by disease recurrence occur earlier than death (Birgisson et al., 2011; Han et al., 2014). All four groups of GBM patients were subjected to DEA (log_2_FC ≥ |0.57|), which revealed more than 400 genes that were inversely regulated among two comparisons (Supplementary Figure S2). Functional annotation of these genes suggested that patients differ in, e.g., response to axon injury, locomotory behavior, synapse organization, and myeloid cell differentiation (Supplementary Figure S2). Not only the relationship between neurodevelopmental and injury programs has recently emerged as a crucial factor influencing treatment sensitivity, but also the injury responses may act as supplementary drivers of phenotypic heterogeneity (Brooks et al., 2022). Pathways associated with locomotory behavior were highly expressed in high-risk GBM patients (Wang et al., 2016). Synapse organization is related to a so-called synapse score showing the prominent ability to predict glioma prognosis, diagnosis, and grading (Ji et al., 2020). Various types of connectivity in glioma (e.g., neuron-to-glioblastoma or glioblastoma-to-glioblastoma) facilitate membrane depolarization followed by calcium transients in networks of tumor cells (Monje, 2020). The inclusion of myeloid cell differentiation in functional analysis suggests that groups of patients may differ in the context of myeloid-derived suppressor cells (MDSCs) that accumulate in late-stage glioblastoma and repress immune activation (Eisenbarth and Wang, 2023a).

MDSCs-related annotations in gene ontology motivated us to perform subsidiary analysis. Although more sophisticated data are required to evaluate MDSCs level among established groups of patients, the estimations obtained via TIMER 2.0 (Supplementary Figure S3) suggested that the immune infiltration by natural killer (NK) cells and myeloid dendritic cells (DCs) was reduced in “Vec”-like “UTU” and “WWOX”-like “DBTRG” groups that include patients having unfavorable survival compared to other two groups. MDSCs are known to hinder the activity of NK cells, the latter being utilized in GBM immunotherapy owing to its ability to counteract tumor progression via cancer cell lysis (Bayik et al., 2020; Breznik et al., 2022; Hosseinalizadeh et al., 2022). Therefore, it is unsurprising that MDSCs are targeted to enhance NK-based treatment (Joshi and Sharabi, 2022). Regarding DCs, their reduction among cancer patients may be due to the preferential differentiation of MDSCs since these two cell types share a common progenitor (Ostrand-Rosenberg et al., 2012). Being a crucial cell type responsible for antigen processing and presenting, DCs initiate anti-tumor immune responses and are used as vaccines in cancer therapy (Srivastava et al., 2019; Zhou et al., 2023). Other immune components with various infiltration levels among established patient groups include the cancer-associated fibroblasts and CD4^+^ Th2 T-cells, summarized in Supplementary Figure S3 (detailed TIMER 2.0 estimations available in Supplementary Table S6). Additionally, genes implicated in the myeloid-associated transcriptional states or accompanying phenomena were collected from the literature (Gangoso et al., 2021; Ravi et al., 2022; Rajendran et al., 2023) and investigated for expression changes between cell lines-resembling groups. Among differentially-expressed genes (Supplementary Figure S3), it is worth mentioning that *APOC1*, *CCL2*, *CCL3*, *IL10*, *IRF8*, *NF1*, and *SPP1* significantly distinguished “UTU” groups of patients (“DBTRG” groups had various *CCL3* expression and might differ in *NF1* level). Except for *NF1*, a tumor suppressor gene that diminishes GBM angiogenesis and migration, the remaining genes were elevated in “Vec”-like “UTU” patients. Loss of *NF1* was found to be correlated with increased immune infiltration within the tumor microenvironment and may prime cells for malignant transformation (Gangoso et al., 2021). *CCL2* was increased in mesenchymal GBM and recruits MDSCs (Chang et al., 2016; Rajendran et al., 2023). Another member from the same chemokine family, *CCL3*, is probably responsible for chemotaxis and is expressed in activated microglia cells that stimulate glioma progression and development (Geribaldi-Doldan et al., 2020; Ravi et al., 2022; Rajendran et al., 2023). Furthermore, *IRF8* contributes to immune evasion (Gangoso et al., 2021), *APOC1* promotes GBM tumorigenesis and is involved in lipid metabolism or immunosuppression (Zheng et al., 2022; Rajendran et al., 2023), whereas *SPP1* is a marker of glioma-associated macrophages that ensures immunosuppression and worsens survival (Rajendran et al., 2023). Lastly, *IL10* is a driver force of tumor immune escape; this interleukin is released by a subset of myeloid cells localized in mesenchymal-like tumor regions (Ravi et al., 2022). Based on the presented subsidiary analysis of immune cellular components and myeloid-associated genes, it can be suggested that groups of patients having unfavorable DFS, especially “Vec”-like “UTU” patients, are affected by more immunosuppressive GBM tumors. Evading immunosurveillance or immunotherapy resistance are major obstacles to GBM treatment (Liu et al., 2021), and dynamic communication with the immune tumor microenvironment is inherent during GBM progression (Arrieta et al., 2023; Guo and Wang, 2023).

### 3.5. Some expression profile differences in the network established from cell lines data were confirmed in patients, revealing genes whose function can explain diverse clinical outcomes

**Figure 5:**
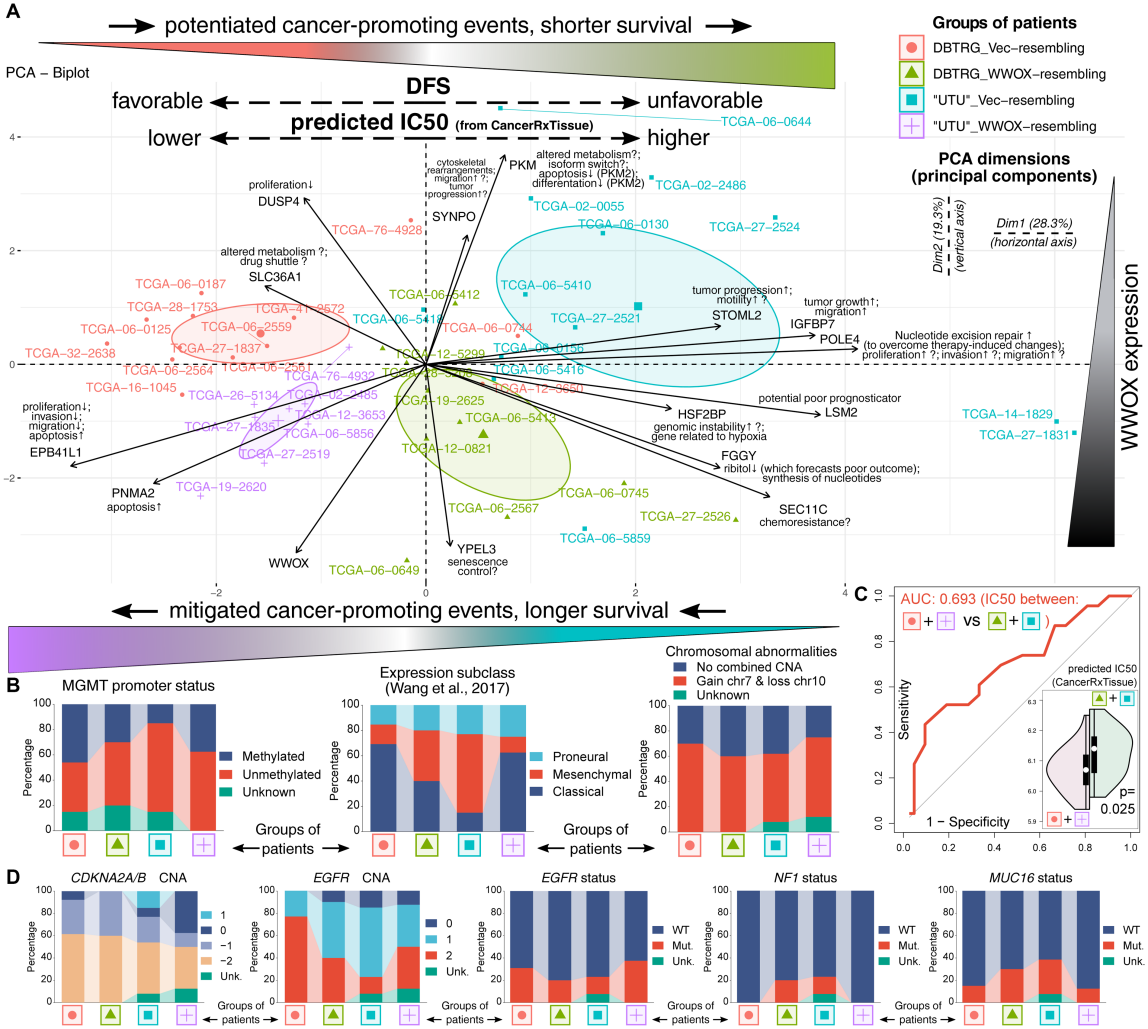
Investigating the *WWOX*-dependent expression profile observed in cell lines using data from patients. **(A)** Principal component analysis indicated a separate cluster for each cell lines-resembling group of patients (see top-right corner for the legend). Incorporating data from survival analysis (groups are colored as in Figure 4C) revealed that patients with more favorable DFS occupied the left side of the graph (red and purple groups), whereas individuals with shorter DFS were located on the right side of the graph (green and blue groups). Moreover, patients occupying the top and bottom sides of the graph differed in *WWOX* expression (the top side corresponds to blue and red groups, whereas the bottom side corresponds to purple and green groups). **(B)** Supplementary data were provided for each group, indicating that, e.g., groups with longer DFS were characterized by more prevalent *MGMT* promoter methylation. This is complemented with **(C)** predicted temozolomide IC50 values acquired from the CancerRxTissue repository that indicate a statistically significant lower TMZ IC50 in groups with longer DFS, suggesting that these patients are better responders to chemotherapy than those with unfavorable DFS. **(D)** Mutation and copy number alteration (CNA) data revealed that patients characterized by worse outcomes had more frequent *MUC16* and *NF1* mutation but less frequent *EGFR* amplification and mutation. Unk, unknown; Mut, mutated; WT, wild-type.

Ultimately, it was decided to utilize the results of differential expression analysis performed on patients and identify which genes demonstrate the cell lines-resembling expression profile change. Alongside *WWOX*/*POLE4*/*HSF2BP*, twelve genes from the network exhibited the expression profile that is consistent between patients and cell lines. These genes were *DUSP4* (Prabhakar et al., 2014), *SLC36A1* (Schaffenrath et al., 2021; Wei et al., 2022), *EPB41L1* (Han et al., 2019), *PNMA2* (Alhajala et al., 2018), *YPEL3* (Phoa et al., 2020), *SEC11C* (Pernia et al., 2020), *FGGY* (Salzillo et al., 2016; Smith et al., 2021), *LSM2* (Sun et al., 2022), *IGFBP7* (Jiang et al., 2008), *STOML2* (Qu et al., 2019; Ma et al., 2021), *PKM* (Mukherjee et al., 2013; El Atat et al., 2023), and *SYNPO* (Ning et al., 2021; Krishna et al., 2023). Some of them were shortly described in previous sections, but the possible roles of all genes are recapitulated in Figure 5A, where the results of PCA are also presented. At first glance, a separate cluster for each group of patients was visible, suggesting that individuals are characterized by distinct expression profile that appears to contribute to patients’ survival. Namely, patients from both comparisons with more favorable DFS occupied the left side of the graph (red and purple groups are in the area with negative values on Dim1, i.e., on the left relative to Dim2). In contrast, individuals with shorter DFS were located on the right side of the graph (green and blue groups are in the area with positive values on Dim1, i.e., on the right relative to Dim2). To facilitate interpretation, groups were marked with the same colors as in Figure 4C. Moreover, patients occupying the top and bottom sides of the graph differed regarding *WWOX* expression (the top side corresponds to blue and red groups, whereas the bottom side corresponds to purple and green groups). Once these groups of patients are considered as “Vec”-like and “WWOX”-like equivalents of cellular variants, the difference between “Vec” and “WWOX” for “UTU”-resembling patients is directed diagonally to the left from top to bottom. In contrast, the difference between “Vec” and “WWOX” for DBTRG-resembling patients is directed diagonally to the right from top to bottom. This suggests that contrary to “UTU” cell lines, *WWOX* overexpression in DBTRG-05MG resulted in distinct expression profile change, resembling that of patients from *WWOX*↑ *POLE4*↑ *HSF2BP*↑ group characterized by inferior outcome presumably due to the events that support cancer development. The latter can be supported by the function of genes included in PCA and their contribution to grouping patients in the first two principal components. Differences in the expression of these genes are also summarized in Supplementary Figure S4 for “UTU”-resembling patients and Supplementary Figure S5 for individuals resembling DBTRG-05MG cellular variants. Lastly, supplementary clinical and molecular data (Cerami et al., 2012; Brennan et al., 2013; Wang et al., 2017; Li et al., 2021; Zakharova et al., 2022) have indicated that groups of patients occupying the right side of the graph (green and blue groups) were characterized by less prevalent Methylguanine methyltransferase (*MGMT*) promoter methylation or more frequent mesenchymal subtype of GBM (Figure 5B). It may explain shorter DFS in these groups since unmethylated *MGMT* promoter predicts an unfavorable outcome (Riemenschneider et al., 2010). Likewise, mesenchymal glioblastoma is the most malignant disease subtype with a signature that forecasts worse survival (Gangoso et al., 2021; Kim et al., 2021). Moreover, higher TMZ IC50 values were noted in the above-mentioned groups in comparison to the other two groups (Figure 5C), suggesting that patients occupying the right side of the graph (green and blue groups) are worse responders to chemotherapy. The groups of patients were not different regarding the Isocitrate dehydrogenase (IDH) status (only two samples were IDH-mutant) or chromosome 1p/19q co-deletion. However, there were some differences in copy number alterations (CNAs) or mutational status among the most frequently affected genes (Supplementary Table S7; Figure 5D). For example, patients characterized by worse outcomes had more frequent *MUC16* mutation and the presence of *NF1* mutation. Alterations of *MUC16* in GBM are associated with unfavorable prognosis (Ferrer, 2023), whereas the *NF1* mutation is a marker of treatment-resistant gliomas (Basindwah et al., 2022). Even though *CDKN2A/B* deletion predicts worse survival (Hsu et al., 2022), CNA data among PCA-included patients are inconclusive. However, *EGFR* amplification and mutation are less frequent in green and blue groups, possibly contributing to their inferior outcomes (Higa et al., 2023) even though some literature data on its prognostic value are debatable (Hobbs et al., 2012; Li et al., 2018a; Alnahhas et al., 2021) or indicate the lack of influence on survival (Ma et al., 2020).

The role of *POLE4* or *HSF2BP* in glioblastoma is poorly understood. Even though some CpG sites functional in high-grade IDH wildtype glioma are located in the genomic location of *POLE4* (Kessler et al., 2020), this gene was only mentioned as being implicated in nuclear excision repair (NER) (Bady et al., 2018). Interestingly, NER is a process by which a wide variety of structurally unrelated DNA lesions generated by chemotherapy and radiotherapy can be removed (Rominiyi and Collis, 2022), suggesting that *POLE4* might control the treatment resistance of GBM. Outside the glioblastoma context, *POLE4* was found to regulate proliferation, migration, and invasion of non-small cell lung cancer (Wang F. et al., 2021). The only GBM-related study mentioning *HSF2BP* investigated hypoxia signatures since the hypoxic interiors of glioblastoma hamper chemotherapy and drive angiogenesis, cell death inhibition, or tumor immune escape (Hu et al., 2012; Lathia et al., 2015; Yaghi et al., 2016; Monteiro et al., 2017). In the mentioned study, *HSF2BP* was upregulated in hypoxia-induced GBM, but no follow-up was performed for this gene (Bhushan et al., 2021). Nevertheless, a study on lung adenocarcinoma indicated that *HSF2BP* forecasts unfavorable patients’ survival and promotes genomic instability (Huang et al., 2021). It is worth noting that such instability may be driven by tumor hypoxia (Tang et al., 2021), which could be considered a valuable hint for future research.

Summarizing all results from this section, it appears that even such a narrow set of genes is able to present differences among cell lines-resembling groups of patients that conforms to discrepancies between DBTRG-05MG and other cell lines found in our previous study. Out of several biological processes investigated *in vitro*, changes in DBTRG-05MG concerned proliferation and tumor growth that were accelerated following *WWOX* overexpression (Kaluzinska-Kolat et al., 2023). Some of the PCA-included genes (*DUSP4*, *EPB41L1*, *IGFBP7*) orchestrate these processes in GBM (Jiang et al., 2008; Prabhakar et al., 2014; Han et al., 2019); it remains to be elucidated whether *POLE4* also regulates glioblastoma proliferation as it does in lung cancer (Wang F. et al., 2021). Nonetheless, it should not be forgotten that the elaborated resemblance of findings from GBM cell lines using data from GBM patients is imperfect, and it cannot be assumed that *POLE4* and *HSF2BP* levels were elevated in patients with unfavorable outcomes due to the higher levels of *WWOX*. However, it highlights that a specific group of patients (6–7% of the entire cohort) cannot benefit from relatively high expression of this tumor suppressor. A possible explanation sheds light on the accessibility of *WWOX* for its protein partners, which seems crucial for the manifestation of pleiotropic functions. Available literature data indicate that two cancer-promoting proteins, TMEM207 and VOPP1, can restrain the antineoplastic activity of WWOX by inhibiting its ability to associate with some partners (Bonin et al., 2018; Bunai et al., 2018). Taouis et al. admitted that this could explain the existence of cancerous cells expressing high levels of *WWOX* (Taouis et al., 2021). Perhaps a similar situation is present in patients with *WWOX*↑ *POLE4*↑ *HSF2BP*↑ profile. Even though *POLE4* and *HSF2BP* were not confirmed to be direct WWOX interactors, their overexpression might upregulate other proteins that hinder the accessibility of WWOX for its cooperators, which is crucial for the maintenance of anti-cancer activity. Further studies are highly encouraged, which should add new dimensions to our current understanding of WWOX multimodality.

### 3.6. Study limitations

Similar to other research, our study has some limitations. For instance, the conclusions drawn from *in vitro* experiments may not accurately reflect the conditions within an organism. It is advisable to conduct a further examination, especially since the data from TCGA-GBM patients included in the current study are encouraging. Moreover, our *in vitro* findings are based on “classical” commercially available cell lines instead of, e.g., glioma stem cells. Although cell lines established and maintained in serum-free neural stem cell media are increasingly used as experimental glioma models (Allen et al., 2016), they resemble but do not mimic original tumors (Garcia-Romero et al., 2016). Compared to conventional cell lines, they are still superior in retaining tumor-specific phenotypes but at the cost of throughput and overall complexity or standardization (Pollard et al., 2009; Boccellato and Rehm, 2022). Given the execution of CAGE-Seq in biological triplicate for each cellular variant, it was inevitable in this study to use more conventional models; however, this can facilitate the replication of the study due to the availability and inexpensiveness. It is also worth mentioning that one of the experimental models utilized in our research – U87MG – can be considered a “problematic” cell line. Historically, the original U87MG cell line was established in 1968 at the University of Uppsala (Ponten and Macintyre, 1968) and is currently denoted as “U87MG Uppsala” according to the Cellosaurus repository (accession number: CVCL_GP63). Herein, we used the cell line that is distributed by most biobanks such as American Type Culture Collection (ATCC) or ECACC (denoted as “U87MG” or “U87MG ATCC”; Cellosaurus accession number: CVCL_0022), which is a different cell line of unknown patient origin but with confirmed central nervous system origin (Allen et al., 2016). It has also been stated that studies utilizing this cell line still reflect brain cancer biology, and there is no need to relegate them (Dolgin, 2016), especially since U87MG has been used in thousands of publications, contributing to the scientific advancement in glioblastoma research (Pokorna et al., 2021). Focusing on discrepancies between DBTRG-05MG versus the other three cell lines can also be considered a limitation since there is a possibility that some valuable results from other comparison schemes were omitted. However, the findings from our previous research revealed that such a scheme is the most justified (Kaluzinska-Kolat et al., 2023). Another aspect is related to the TCGA-GBM cohort that, despite being one of the most considerable resources of glioblastoma patients’ data, does not provide transcriptomic profiling for all individuals. This has complicated the stratification based on the *WWOX*/*POLE4*/*HSF2BP* level and limited the number of genes that were used to establish expression profiles. Lastly, provided explanations for some results (such as those related to immune cellular components and myeloid-associated genes) may require further verification using dedicated and expensive methods that are currently out of our reach but which will be undertaken in the future. Still, these explanations may aid the scientific community in choosing subsequent research directions.

## 4. Conclusion

The following inferences can be drawn from consecutive parts of this study. The expression profile of DBTRG-05MG is distinct from U87MG, T98G, and U251MG; some of the DBTRG-05MG driver genes are unique for this cell line (*FGGY*, *NCBP2AS2*, *UBTD1*) or may be present in other cell lines but are regulated inversely following *WWOX* overexpression (*CARHSP1*, *DUSP4*, *FOSL2*, *HSF2BP*, *LINC01547*, *PLCD3*, *POLE4*, *RUSC1*, *SH3BP2*, *SLC36A1*, *SLCO4A1*, *THAP11*, *ZNF786*). Drivers, top hubs, and other *WWOX*-dependent genes identified in this study regulate various processes, including those important for the nervous system. Genes that were not previously the subject of GBM-related research (e.g., *FGGY*, *LINC01547*, *NCBP2AS2*, *DYM*, *GNPDA1*, *GPS1*, *GET4*) warrant further investigation. Some driver genes with consistent expression change following *WWOX* overexpression were found in all cell lines (*PRKRA*, *PNMA2*, *GPS1*, *ARF3*), suggesting that a part of dependent processes might be regulated similarly in U87MG, T98G, U251MG, and DBTRG-05MG, which was the case in our previous study. Constructing the network has enabled the extension outside the drivers-related context, which showed other genes that may be inversely regulated in DBTRG-05MG relative to other cell lines. When split into relevant parts, functional annotation of the network suggested that processes such as apoptosis may be controlled congruously between cell lines, whereas microglia proliferation or neurofibrillary tangle assembly might distinguish DBTRG-05MG. Subsequently, it was possible to identify patients resembling cell line comparisons using *WWOX*, *POLE4*, and *HSF2BP* data, whose expression in various combinations indicated, e.g., distinct survival (OS and DFS), sensitivity to TMZ, or infiltration of immune cellular components. Some gene expression differences between cell lines were reflected in patients; genes manifesting equivalent profiles were *DUSP4*, *SLC36A1*, *EPB41L1*, *PNMA2*, *YPEL3*, *SEC11C*, *FGGY*, *LSM2*, *IGFBP7*, *STOML2*, *PKM*, and *SYNPO*. Their contribution to biological processes seems to clarify the cancer-related events that determined longer or shorter survival. The role of *POLE4* or *HSF2BP* in GBM is poorly understood but may be related to nuclear excision repair that renders treatment resistance or might affect genomic instability, presumably in hypoxic interiors. It has been proposed that in patients with *WWOX*↑ *POLE4*↑ *HSF2BP*↑ expression profile (who account for ~6–7% of the entire cohort), WWOX protein may not be available for its cooperating partners, which is manifested by the absence of anti-cancer activity despite a high *WWOX* level. Such a circumstance was previously found in the literature but not in the case of glioblastoma. Whether POLE4 and HSF2BP bind WWOX directly is yet to be elucidated in future research, where particular attention should be drawn to their role and interaction networks in GBM.

All things considered, data from cell lines enabled the identification of the group of patients among which a high *WWOX* expression with simultaneously elevated expression of other genes (herein: *POLE4* and *HSF2BP*) did not bring prognostic benefits and may be related to the more cancer-promoting profile. This group accounts for about 6–7% of the investigated cohort, which translates into a considerable percentage on a global scale and may complement the qualification of patients for personalized medicine in the future.

## Data availability statement

The datasets presented in this study can be found in online repositories. The names of the repository/repositories and accession number(s) can be found in the article/Supplementary material.

## Author contributions

ŻK-K: Conceptualization, Methodology, Visualization, Writing – original draft, Writing – review & editing. DK: Methodology, Visualization, Writing – review & editing. KK: Methodology, Writing – review & editing. EP: Writing – review & editing. AB: Conceptualization, Methodology, Supervision, Writing – review & editing.

## Funding

The author(s) declare financial support was received for the research, authorship, and/or publication of this article. This research was funded by both the National Science Centre of Poland (grant number: 2021/41/N/NZ2/01375) and the Medical University of Lodz (grant number: 503/0-072-02/503-01-001). The funding bodies had no role in the study design, collection, analysis, and interpretation of data and in writing the manuscript.

## Conflict of interest

The authors declare that the research was conducted in the absence of any commercial or financial relationships that could be construed as a potential conflict of interest.

The author(s) declared that they were an editorial board member of Frontiers, at the time of submission. This had no impact on the peer review process and the final decision.

## Publisher’s note

All claims expressed in this article are solely those of the authors and do not necessarily represent those of their affiliated organizations, or those of the publisher, the editors and the reviewers. Any product that may be evaluated in this article, or claim that may be made by its manufacturer, is not guaranteed or endorsed by the publisher.

## References

[ref1] Afshar Moghaddam N. Mahsuni P. Taheri D. (2015). Evaluation of endoglin as an angiogenesis marker in glioblastoma. Iran. J. Pathol. 10, 89–96. doi: 10.7508/ijp.2015.02.002 26351468 PMC4539765

[ref2] Al Seesi S. Tiagueu Y. T. Zelikovsky A. Mandoiu I. I. (2014). Bootstrap-based differential gene expression analysis for RNA-Seq data with and without replicates. BMC Genomics 15:S2. doi: 10.1186/1471-2164-15-S8-S2 PMC4248812 25435284

[ref3] Alhajala H. S. Nguyen H. S. Shabani S. Best B. Kaushal M. Al-Gizawiy M. M.. (2018). Irradiation of pediatric glioblastoma cells promotes radioresistance and enhances glioma malignancy via genome-wide transcriptome changes. Oncotarget 9, 34122–34131. doi: 10.18632/oncotarget.26137 30344926 PMC6183347

[ref4] Ali M. W. Patro C. P. K. Zhu J. J. Dampier C. H. Plummer S. J. Kuscu C.. (2021). A functional variant on 20q13.33 related to glioma risk alters enhancer activity and modulates expression of multiple genes. Hum. Mutat. 42, 77–88. doi: 10.1002/humu.24134 33169458 PMC7839675

[ref5] Allen M. Bjerke M. Edlund H. Nelander S. Westermark B. (2016). Origin of the U87MG glioma cell line: good news and bad news. Sci. Transl. Med. 8:354re353. doi: 10.1126/scitranslmed.aaf6853 27582061

[ref6] Almeida-Silva F. Venancio T. M. (2022). BioNERO: an all-in-one R/Bioconductor package for comprehensive and easy biological network reconstruction. Funct. Integr. Genomics 22, 131–136. doi: 10.1007/s10142-021-00821-9 34787733

[ref7] Alnahhas I. Rayi A. Guillermo Prieto Eibl M. D. P. Ong S. Giglio P. Puduvalli V. (2021). Prognostic implications of epidermal and platelet-derived growth factor receptor alterations in 2 cohorts of IDHwt glioblastoma. Neurooncol. Adv. 3:vdab127. doi: 10.1093/noajnl/vdab127 34667950 PMC8519397

[ref8] Arrieta V. A. Dmello C. McGrail D. J. Brat D. J. Lee-Chang C. Heimberger A. B.. (2023). Immune checkpoint blockade in glioblastoma: from tumor heterogeneity to personalized treatment. J. Clin. Invest. 133:e163447. doi: 10.1172/JCI163447 36647828 PMC9843050

[ref9] Atkins I. Kinnersley B. Ostrom Q. T. Labreche K. Il'yasova D. Armstrong G. N.. (2019). Transcriptome-wide association study identifies new candidate susceptibility genes for glioma. Cancer Res. 79, 2065–2071. doi: 10.1158/0008-5472.CAN-18-2888 30709929 PMC6522343

[ref10] Bady P. Kurscheid S. Delorenzi M. Gorlia T. van den Bent M. J. Hoang-Xuan K.. (2018). The DNA methylome of DDR genes and benefit from RT or TMZ in IDH mutant low-grade glioma treated in EORTC 22033. Acta Neuropathol. 135, 601–615. doi: 10.1007/s00401-018-1810-6 29368212 PMC5978935

[ref11] Basindwah S. Alkhalidi H. Abdelwarith A. Elwatidy S. (2022). Ten-year survival in glioblastoma patient with neurofibromatosis type 1: illustrative case. J. Neurosurg. Case. Less. 3:CASE21630. doi: 10.3171/CASE21630 PMC9379713 36130570

[ref12] Bastos A. G. P. Carvalho B. Silva R. Leitao D. Linhares P. Vaz R.. (2022). Endoglin (CD105) and proliferation index in recurrent glioblastoma treated with anti-angiogenic therapy. Front. Oncol. 12:910196. doi: 10.3389/fonc.2022.910196 36147918 PMC9486379

[ref13] Bayik D. Zhou Y. Park C. Hong C. Vail D. Silver D. J.. (2020). Myeloid-derived suppressor cell subsets drive glioblastoma growth in a sex-specific manner. Cancer Discov. 10, 1210–1225. doi: 10.1158/2159-8290.CD-19-1355 32300059 PMC7415660

[ref14] Bergwerff M. Verberne M. E. DeRuiter M. C. Poelmann R. E. Gittenberger-de Groot A. C. (1998). Neural crest cell contribution to the developing circulatory system: implications for vascular morphology? Circ. Res. 82, 221–231. doi: 10.1161/01.res.82.2.221 9468193

[ref15] Bhushan A. Kumari R. Srivastava T. (2021). Scouting for common genes in the heterogenous hypoxic tumor microenvironment and their validation in glioblastoma. 3 Biotech 11:451. doi: 10.1007/s13205-021-02987-2 PMC8473528 34631352

[ref16] Birgisson H. Wallin U. Holmberg L. Glimelius B. (2011). Survival endpoints in colorectal cancer and the effect of second primary other cancer on disease free survival. BMC Cancer 11:438. doi: 10.1186/1471-2407-11-438 21989154 PMC3209454

[ref17] Birzu C. French P. Caccese M. Cerretti G. Idbaih A. Zagonel V.. (2020). Recurrent glioblastoma: from molecular landscape to new treatment perspectives. Cancers 13:47. doi: 10.3390/cancers13010047 33375286 PMC7794906

[ref18] Blainey P. Krzywinski M. Altman N. (2014). Points of significance: replication. Nat. Methods 11, 879–880. doi: 10.1038/nmeth.3091 25317452

[ref19] Blakstad H. Brekke J. Rahman M. A. Arnesen V. S. Miletic H. Brandal P.. (2023). Survival in a consecutive series of 467 glioblastoma patients: association with prognostic factors and treatment at recurrence at two independent institutions. PLoS One 18:e0281166. doi: 10.1371/journal.pone.0281166 36730349 PMC9894455

[ref20] Boccellato C. Rehm M. (2022). Glioblastoma, from disease understanding towards optimal cell-based in vitro models. Cell. Oncol. 45, 527–541. doi: 10.1007/s13402-022-00684-7 PMC9424171 35763242

[ref21] Bonin F. Taouis K. Azorin P. Petitalot A. Tariq Z. Nola S.. (2018). VOPP1 promotes breast tumorigenesis by interacting with the tumor suppressor WWOX. BMC Biol. 16:109. doi: 10.1186/s12915-018-0576-6 30285739 PMC6169085

[ref22] Brennan C. W. Verhaak R. G. McKenna A. Campos B. Noushmehr H. Salama S. R.. (2013). The somatic genomic landscape of glioblastoma. Cells 155, 462–477. doi: 10.1016/j.cell.2013.09.034 PMC3910500 24120142

[ref23] Breznik B. Ko M. W. Tse C. Chen P. C. Senjor E. Majc B.. (2022). Infiltrating natural killer cells bind, lyse and increase chemotherapy efficacy in glioblastoma stem-like tumorospheres. Commun. Biol. 5:436. doi: 10.1038/s42003-022-03402-z 35538218 PMC9090761

[ref24] Brooks L. J. Simpson Ragdale H. Hill C. S. Clements M. Parrinello S. (2022). Injury programs shape glioblastoma. Trends Neurosci. 45, 865–876. doi: 10.1016/j.tins.2022.08.006 36089406

[ref25] Bunai K. Okubo H. Hano K. Inoue K. Kito Y. Saigo C.. (2018). TMEM207 hinders the tumour suppressor function of WWOX in oral squamous cell carcinoma. J. Cell. Mol. Med. 22, 1026–1033. doi: 10.1111/jcmm.13456 29164763 PMC5783854

[ref26] Casalou C. Ferreira A. Barral D. C. (2020). The role of ARF family proteins and their regulators and effectors in cancer progression: a therapeutic perspective. Front. Cell Dev. Biol. 8:217. doi: 10.3389/fcell.2020.00217 32426352 PMC7212444

[ref27] Cerami E. Gao J. Dogrusoz U. Gross B. E. Sumer S. O. Aksoy B. A.. (2012). The cBio cancer genomics portal: an open platform for exploring multidimensional cancer genomics data. Cancer Discov. 2, 401–404. doi: 10.1158/2159-8290.CD-12-0095 22588877 PMC3956037

[ref28] Chang A. L. Miska J. Wainwright D. A. Dey M. Rivetta C. V. Yu D.. (2016). CCL2 produced by the glioma microenvironment is essential for the recruitment of regulatory T cells and myeloid-derived suppressor cells. Cancer Res. 76, 5671–5682. doi: 10.1158/0008-5472.CAN-16-0144 27530322 PMC5050119

[ref29] Chiang M. F. Chou P. Y. Wang W. J. Sze C. I. Chang N. S. (2013). Tumor suppressor WWOX and p53 alterations and drug resistance in glioblastomas. Front. Oncol. 3:43. doi: 10.3389/fonc.2013.00043 23459853 PMC3586680

[ref30] Chiang M. F. Yeh S. T. Liao H. F. Chang N. S. Chen Y. J. (2012). Overexpression of WW domain-containing oxidoreductase WOX1 preferentially induces apoptosis in human glioblastoma cells harboring mutant p53. Biomed. Pharmacother. 66, 433–438. doi: 10.1016/j.biopha.2012.03.003 22898080

[ref31] Chong Z. X. Ho W. Y. Yeap S. K. (2023). Delineating the tumour-regulatory roles of EYA4. Biochem. Pharmacol. 210:115466. doi: 10.1016/j.bcp.2023.115466 36849065

[ref32] Chow K. H. Park H. J. George J. Yamamoto K. Gallup A. D. Graber J. H.. (2017). S100A4 is a biomarker and regulator of glioma stem cells that is critical for mesenchymal transition in glioblastoma. Cancer Res. 77, 5360–5373. doi: 10.1158/0008-5472.CAN-17-1294 28807938 PMC5626628

[ref33] Conway J. R. Lex A. Gehlenborg N. (2017). UpSetR: an R package for the visualization of intersecting sets and their properties. Bioinformatics 33, 2938–2940. doi: 10.1093/bioinformatics/btx364 28645171 PMC5870712

[ref34] Delgado-Martin B. Medina M. A. (2020). Advances in the knowledge of the molecular biology of glioblastoma and its impact in patient diagnosis, stratification, and treatment. Adv. Sci. 7:1902971. doi: 10.1002/advs.201902971 PMC7201267 32382477

[ref35] Dobin A. Davis C. A. Schlesinger F. Drenkow J. Zaleski C. Jha S.. (2013). STAR: ultrafast universal RNA-seq aligner. Bioinformatics 29, 15–21. doi: 10.1093/bioinformatics/bts635 23104886 PMC3530905

[ref36] Dolgin E. (2016). Venerable brain-cancer cell line faces identity crisis. Nature 537, 149–150. doi: 10.1038/nature.2016.20515 27604929

[ref37] Doncheva N. T. Morris J. H. Gorodkin J. Jensen L. J. (2019). Cytoscape StringApp: network analysis and visualization of proteomics data. J. Proteome Res. 18, 623–632. doi: 10.1021/acs.jproteome.8b00702 30450911 PMC6800166

[ref38] Dong S. Hou D. Peng Y. Chen X. Li H. Wang H. (2022). Pan-cancer analysis of the prognostic and immunotherapeutic value of MITD1. Cells 11:3308. doi: 10.3390/cells11203308 36291174 PMC9600621

[ref39] Dymova M. A. Kuligina E. V. Richter V. A. (2021). Molecular mechanisms of drug resistance in glioblastoma. Int. J. Mol. Sci. 22:6385. doi: 10.3390/ijms22126385 34203727 PMC8232134

[ref40] Eisenbarth D. Wang Y. A. (2023a). Glioblastoma heterogeneity at single cell resolution. Oncogene 42, 2155–2165. doi: 10.1038/s41388-023-02738-y 37277603 PMC10913075

[ref41] Eisenbarth D. Wang Y. A. (2023b). Insights into the co-evolution of glioblastoma and associated macrophages. J. Cancer Metastasis Treat. 9:14. doi: 10.20517/2394-4722.2023.09

[ref42] El Atat O. Naser R. Abdelkhalek M. Habib R. A. El Sibai M. (2023). Molecular targeted therapy: a new avenue in glioblastoma treatment. Oncol. Lett. 25:46. doi: 10.3892/ol.2022.13632 36644133 PMC9811647

[ref43] Fedele M. Cerchia L. Pegoraro S. Sgarra R. Manfioletti G. (2019). Proneural-mesenchymal transition: phenotypic plasticity to acquire multitherapy resistance in glioblastoma. Int. J. Mol. Sci. 20:2746. doi: 10.3390/ijms20112746 31167470 PMC6600373

[ref44] Ferrer V. P. (2023). MUC16 mutation is associated with tumor grade, clinical features, and prognosis in glioma patients. Cancer Genet. 270-271, 22–30. doi: 10.1016/j.cancergen.2022.11.003 36436416

[ref45] Forbes S. A. Bindal N. Bamford S. Cole C. Kok C. Y. Beare D.. (2011). COSMIC: mining complete cancer genomes in the catalogue of somatic mutations in cancer. Nucleic Acids Res. 39, D945–D950. doi: 10.1093/nar/gkq929 20952405 PMC3013785

[ref46] Gangoso E. Southgate B. Bradley L. Rus S. Galvez-Cancino F. McGivern N.. (2021). Glioblastomas acquire myeloid-affiliated transcriptional programs via epigenetic immunoediting to elicit immune evasion. Cells 184, 2454–2470. doi: 10.1016/j.cell.2021.03.023 PMC8099351 33857425

[ref47] Garcia-Romero N. Gonzalez-Tejedo C. Carrion-Navarro J. Esteban-Rubio S. Rackov G. Rodriguez-Fanjul V.. (2016). Cancer stem cells from human glioblastoma resemble but do not mimic original tumors after in vitro passaging in serum-free media. Oncotarget 7, 65888–65901. doi: 10.18632/oncotarget.11676 27589567 PMC5323200

[ref48] Geribaldi-Doldan N. Fernandez-Ponce C. Quiroz R. N. Sanchez-Gomar I. Escorcia L. G. Velasquez E. P.. (2020). The role of microglia in glioblastoma. Front. Oncol. 10:603495. doi: 10.3389/fonc.2020.603495 33585220 PMC7879977

[ref49] Ghandi M. Huang F. W. Jane-Valbuena J. Kryukov G. V. Lo C. C. McDonald E. R. 3rd. (2019). Next-generation characterization of the cancer cell line Encyclopedia. Nature 569, 503–508. doi: 10.1038/s41586-019-1186-3 31068700 PMC6697103

[ref50] Granger B. R. Chang Y. C. Wang Y. DeLisi C. Segre D. Hu Z. (2016). Visualization of metabolic interaction networks in microbial communities using VisANT 5.0. PLoS Comput. Biol. 12:e1004875. doi: 10.1371/journal.pcbi.1004875 27081850 PMC4833320

[ref51] Guo X. Wang G. (2023). Advances in research on immune escape mechanism of glioma. CNS Neurosci. Ther. 29, 1709–1720. doi: 10.1111/cns.14217 37088950 PMC10324367

[ref52] Han K. Ren M. Wick W. Abrey L. Das A. Jin J.. (2014). Progression-free survival as a surrogate endpoint for overall survival in glioblastoma: a literature-based meta-analysis from 91 trials. Neuro-Oncology 16, 696–706. doi: 10.1093/neuonc/not236 24335699 PMC3984546

[ref53] Han X. Wang X. Li H. Zhang H. (2019). Mechanism of microRNA-431-5p-EPB41L1 interaction in glioblastoma multiforme cells. Arch. Med. Sci. 15, 1555–1564. doi: 10.5114/aoms.2019.88274 31749885 PMC6855151

[ref54] Hedna R. Kovacic H. Pagano A. Peyrot V. Robin M. Devred F.. (2022). Tau protein as therapeutic target for cancer? Focus on glioblastoma. Cancers 14:5386. doi: 10.3390/cancers14215386 36358803 PMC9653627

[ref55] Higa N. Akahane T. Hamada T. Yonezawa H. Uchida H. Makino R.. (2023). Distribution and favorable prognostic implication of genomic EGFR alterations in IDH-wildtype glioblastoma. Cancer Med. 12, 49–60. doi: 10.1002/cam4.4939 35695190 PMC9844636

[ref56] Hobbs J. Nikiforova M. N. Fardo D. W. Bortoluzzi S. Cieply K. Hamilton R. L.. (2012). Paradoxical relationship between the degree of EGFR amplification and outcome in glioblastomas. Am. J. Surg. Pathol. 36, 1186–1193. doi: 10.1097/PAS.0b013e3182518e12 22472960 PMC3393818

[ref57] Hopp S. C. Lin Y. Oakley D. Roe A. D. DeVos S. L. Hanlon D.. (2018). The role of microglia in processing and spreading of bioactive tau seeds in Alzheimer's disease. J. Neuroinflammation 15:269. doi: 10.1186/s12974-018-1309-z 30227881 PMC6145371

[ref58] Hosseinalizadeh H. Habibi Roudkenar M. Mohammadi Roushandeh A. Kuwahara Y. Tomita K. Sato T. (2022). Natural killer cell immunotherapy in glioblastoma. Discov. Oncol. 13:113. doi: 10.1007/s12672-022-00567-1 36305981 PMC9616998

[ref59] Hsu E. J. Thomas J. Maher E. A. Youssef M. Timmerman R. D. Wardak Z.. (2022). Impact of CDKN2A/B, MTAP, and TERT genetic alterations on survival in IDH wild type glioblastomas. Discov. Oncol. 13:126. doi: 10.1007/s12672-022-00590-2 36380219 PMC9666584

[ref60] Hu Y. L. DeLay M. Jahangiri A. Molinaro A. M. Rose S. D. Carbonell W. S.. (2012). Hypoxia-induced autophagy promotes tumor cell survival and adaptation to antiangiogenic treatment in glioblastoma. Cancer Res. 72, 1773–1783. doi: 10.1158/0008-5472.CAN-11-3831 22447568 PMC3319869

[ref61] Huang Z. Liu Z. Cheng X. Han Z. Li J. Xia T.. (2021). Prognostic significance of HSF2BP in lung adenocarcinoma. Ann. Transl. Med. 9:1559. doi: 10.21037/atm-21-4935 34790765 PMC8576644

[ref62] Ji X. Zhang H. Cui Q. (2020). A panel of synapse-related genes as a biomarker for gliomas. Front. Neurosci. 14:822. doi: 10.3389/fnins.2020.00822 32848578 PMC7431624

[ref63] Jiang W. Xiang C. Cazacu S. Brodie C. Mikkelsen T. (2008). Insulin-like growth factor binding protein 7 mediates glioma cell growth and migration. Neoplasia 10, 1335–1342. doi: 10.1593/neo.08694 19048112 PMC2586684

[ref64] Joshi S. Sharabi A. (2022). Targeting myeloid-derived suppressor cells to enhance natural killer cell-based immunotherapy. Pharmacol. Ther. 235:108114. doi: 10.1016/j.pharmthera.2022.108114 35122833 PMC9189042

[ref65] Jovcevska I. (2020). Next generation sequencing and machine learning technologies are painting the epigenetic portrait of glioblastoma. Front. Oncol. 10:798. doi: 10.3389/fonc.2020.00798 32500035 PMC7243123

[ref66] Kaluzinska-Kolat Z. Kosla K. Kolat D. Pluciennik E. Bednarek A. K. (2023). Antineoplastic nature of WWOX in glioblastoma is mainly a consequence of reduced cell viability and invasion. Biology 12:465. doi: 10.3390/biology12030465 36979157 PMC10045224

[ref67] Kessler T. Berberich A. Sadik A. Sahm F. Gorlia T. Meisner C.. (2020). Methylome analyses of three glioblastoma cohorts reveal chemotherapy sensitivity markers within DDR genes. Cancer Med. 9, 8373–8385. doi: 10.1002/cam4.3447 32991787 PMC7666733

[ref68] Kim N. Choi W. S. (2011). Proapoptotic role of nuclear clusterin in brain. Anat. Cell Biol. 44, 169–175. doi: 10.5115/acb.2011.44.3.169 22025968 PMC3195820

[ref69] Kim Y. Varn F. S. Park S. H. Yoon B. W. Park H. R. Lee C.. (2021). Perspective of mesenchymal transformation in glioblastoma. Acta Neuropathol. Commun. 9:50. doi: 10.1186/s40478-021-01151-4 33762019 PMC7992784

[ref70] Kiseleva L. N. Kartashev A. V. Vartanyan N. L. Pinevich A. A. Samoilovich M. P. (2016). A172 and T98G cell lines characteristics. Cell Tiss. Biol. 10, 341–348. doi: 10.1134/s1990519x16050072 30188626

[ref71] Kosla K. Kaluzinska Z. Bednarek A. K. (2020). The WWOX gene in brain development and pathology. Exp. Biol. Med. 245, 1122–1129. doi: 10.1177/1535370220924618 PMC7400721 32389029

[ref72] Kosla K. Pluciennik E. Kurzyk A. Jesionek-Kupnicka D. Kordek R. Potemski P.. (2011). Molecular analysis of WWOX expression correlation with proliferation and apoptosis in glioblastoma multiforme. J. Neuro-Oncol. 101, 207–213. doi: 10.1007/s11060-010-0254-1, PMID: 20535528 PMC2996532

[ref73] Krishna S. Choudhury A. Keough M. B. Seo K. Ni L. Kakaizada S.. (2023). Glioblastoma remodelling of human neural circuits decreases survival. Nature 617, 599–607. doi: 10.1038/s41586-023-06036-1 37138086 PMC10191851

[ref74] Kulesa A. Krzywinski M. Blainey P. Altman N. (2015). Sampling distributions and the bootstrap. Nat. Methods 12, 477–478. doi: 10.1038/nmeth.3414 26221652 PMC4737599

[ref75] Lan B. Zhao H. He Y. Zhao Z. Wang N. Gao Y. (2022). Inhibition of human peptide deformylase by actinonin sensitizes glioblastoma cells to temozolomide chemotherapy. Exp. Cell Res. 420:113358. doi: 10.1016/j.yexcr.2022.113358 36116558

[ref76] Langfelder P. Horvath S. (2008). WGCNA: an R package for weighted correlation network analysis. BMC Bioinform. 9:559. doi: 10.1186/1471-2105-9-559 PMC2631488 19114008

[ref77] Lathia J. D. Mack S. C. Mulkearns-Hubert E. E. Valentim C. L. Rich J. N. (2015). Cancer stem cells in glioblastoma. Genes Dev. 29, 1203–1217. doi: 10.1101/gad.261982.115 26109046 PMC4495393

[ref78] Law C. W. Chen Y. Shi W. Smyth G. K. (2014). Voom: precision weights unlock linear model analysis tools for RNA-seq read counts. Genome Biol. 15:R29. doi: 10.1186/gb-2014-15-2-r29 24485249 PMC4053721

[ref79] Lê S. Josse J. Husson F. (2008). FactoMineR: AnRPackage for multivariate analysis. J. Stat. Softw. 25, 1–18. doi: 10.18637/jss.v025.i01

[ref80] Lei Q. Gu H. Li L. Wu T. Xie W. Li M.. (2020). TNIP1-mediated TNF-alpha/NF-kappaB signalling cascade sustains glioma cell proliferation. J. Cell. Mol. Med. 24, 530–538. doi: 10.1111/jcmm.14760, PMID: 31691497 PMC6933386

[ref81] Li J. Liang R. Song C. Xiang Y. Liu Y. (2018a). Prognostic significance of epidermal growth factor receptor expression in glioma patients. Onco. Targets. Ther. 11, 731–742. doi: 10.2147/OTT.S155160 29445288 PMC5808691

[ref82] Li T. Fu J. Zeng Z. Cohen D. Li J. Chen Q.. (2020). TIMER2.0 for analysis of tumor-infiltrating immune cells. Nucleic Acids Res. 48, W509–W514. doi: 10.1093/nar/gkaa407 32442275 PMC7319575

[ref83] Li Y. Umbach D. M. Krahn J. M. Shats I. Li X. Li L. (2021). Predicting tumor response to drugs based on gene-expression biomarkers of sensitivity learned from cancer cell lines. BMC Genomics 22:272. doi: 10.1186/s12864-021-07581-7 33858332 PMC8048084

[ref84] Li Z. Qiu R. Qiu X. Tian T. (2018b). EYA4 promotes cell proliferation through downregulation of p27Kip1 in glioma. Cell. Physiol. Biochem. 49, 1856–1869. doi: 10.1159/000493631, PMID: 30231237

[ref85] Liao Y. Wang J. Jaehnig E. J. Shi Z. Zhang B. (2019). WebGestalt 2019: gene set analysis toolkit with revamped UIs and APIs. Nucleic Acids Res. 47, W199–W205. doi: 10.1093/nar/gkz401 31114916 PMC6602449

[ref86] Liu I. Hack O. A. Filbin M. G. (2021). The imitation game: how glioblastoma outmaneuvers immune attack. Cells 184, 2278–2281. doi: 10.1016/j.cell.2021.04.008 33930294

[ref87] Liu S. Y. Chiang M. F. Chen Y. J. (2015). Role of WW domain proteins WWOX in development, prognosis, and treatment response of glioma. Exp. Biol. Med. 240, 315–323. doi: 10.1177/1535370214561588 PMC4935226 25432984

[ref88] Love M. I. Huber W. Anders S. (2014). Moderated estimation of fold change and dispersion for RNA-seq data with DESeq2. Genome Biol. 15:550. doi: 10.1186/s13059-014-0550-8 25516281 PMC4302049

[ref89] Ma S. Rudra S. Campian J. L. Dahiya S. Dunn G. P. Johanns T.. (2020). Prognostic impact of CDKN2A/B deletion, TERT mutation, and EGFR amplification on histological and molecular IDH-wildtype glioblastoma. Neurooncol. Adv. 2:vdaa126. doi: 10.1093/noajnl/vdaa126 33235995 PMC7668466

[ref90] Ma W. Chen Y. Xiong W. Li W. Xu Z. Wang Y.. (2021). STOML2 interacts with PHB through activating MAPK signaling pathway to promote colorectal cancer proliferation. J. Exp. Clin. Cancer Res. 40:359. doi: 10.1186/s13046-021-02116-0 34781982 PMC8591804

[ref91] Marin-Rubio J. L. Vela-Martin L. Fernandez-Piqueras J. Villa-Morales M. (2019). FADD in cancer: mechanisms of altered expression and function, and clinical implications. Cancers 11:1462. doi: 10.3390/cancers11101462 31569512 PMC6826683

[ref92] Maris C. D'Haene N. Trepant A. L. Le Mercier M. Sauvage S. Allard J.. (2015). IGF-IR: a new prognostic biomarker for human glioblastoma. Br. J. Cancer 113, 729–737. doi: 10.1038/bjc.2015.242 26291053 PMC4559821

[ref93] Marques C. Unterkircher T. Kroon P. Oldrini B. Izzo A. Dramaretska Y.. (2021). NF1 regulates mesenchymal glioblastoma plasticity and aggressiveness through the AP-1 transcription factor FOSL1. elife 10:e64846. doi: 10.7554/eLife.64846 34399888 PMC8370767

[ref94] Metaxas A. Kempf S. J. (2016). Neurofibrillary tangles in Alzheimer's disease: elucidation of the molecular mechanism by immunohistochemistry and tau protein phospho-proteomics. Neural Regen. Res. 11, 1579–1581. doi: 10.4103/1673-5374.193234 27904486 PMC5116834

[ref95] Monje M. (2020). Synaptic communication in brain cancer. Cancer Res. 80, 2979–2982. doi: 10.1158/0008-5472.CAN-20-0646 32381657 PMC7367763

[ref96] Monteiro A. R. Hill R. Pilkington G. J. Madureira P. A. (2017). The role of hypoxia in glioblastoma invasion. Cells 6:45. doi: 10.3390/cells6040045 29165393 PMC5755503

[ref97] Morris J. H. Kuchinsky A. Ferrin T. E. Pico A. R. (2014). enhancedGraphics: a Cytoscape app for enhanced node graphics. F1000Res 3:147. doi: 10.12688/f1000research.4460.1 25285206 PMC4176421

[ref98] Mukherjee J. Phillips J. J. Zheng S. Wiencke J. Ronen S. M. Pieper R. O. (2013). Pyruvate kinase M2 expression, but not pyruvate kinase activity, is up-regulated in a grade-specific manner in human glioma. PLoS One 8:e57610. doi: 10.1371/journal.pone.0057610 23451252 PMC3581484

[ref99] Neftel C. Laffy J. Filbin M. G. Hara T. Shore M. E. Rahme G. J.. (2019). An integrative model of cellular states, plasticity, and genetics for glioblastoma. Cells 178, 835–849 e821. doi: 10.1016/j.cell.2019.06.024 PMC6703186 31327527

[ref100] Ning L. Suleiman H. Y. Miner J. H. (2021). Synaptopodin deficiency exacerbates kidney disease in a mouse model of Alport syndrome. Am. J. Physiol. Renal Physiol. 321, F12–F25. doi: 10.1152/ajprenal.00035.2021 34029143 PMC8321802

[ref101] Ogluszka M. Orzechowska M. Jedroszka D. Witas P. Bednarek A. K. (2019). Evaluate Cutpoints: adaptable continuous data distribution system for determining survival in Kaplan-Meier estimator. Comput. Methods Prog. Biomed. 177, 133–139. doi: 10.1016/j.cmpb.2019.05.023 31319941

[ref102] Ohanian J. Ohanian V. (2001). Lipid second messenger regulation: the role of diacylglycerol kinases and their relevance to hypertension. J. Hum. Hypertens. 15, 93–98. doi: 10.1038/sj.jhh.1001139 11317187

[ref103] Oliveira M. N. Pillat M. M. Baranova J. Andrejew R. dos Santos B. L. Costa S. L.. (2022). Glioblastoma cell invasiveness and epithelial-to-mesenchymal transitioning are modulated by kinin receptors. Adv. Cancer Biol. 4:100045. doi: 10.1016/j.adcanc.2022.100045

[ref104] Oronsky B. Reid T. R. Oronsky A. Sandhu N. Knox S. J. (2020). A review of newly diagnosed glioblastoma. Front. Oncol. 10:574012. doi: 10.3389/fonc.2020.574012 33614476 PMC7892469

[ref105] Ostrand-Rosenberg S. Sinha P. Beury D. W. Clements V. K. (2012). Cross-talk between myeloid-derived suppressor cells (MDSC), macrophages, and dendritic cells enhances tumor-induced immune suppression. Semin. Cancer Biol. 22, 275–281. doi: 10.1016/j.semcancer.2012.01.011 22313874 PMC3701942

[ref106] Osuka S. Zhu D. Zhang Z. Li C. Stackhouse C. T. Sampetrean O.. (2021). N-cadherin upregulation mediates adaptive radioresistance in glioblastoma. J. Clin. Invest. 131:e136098. doi: 10.1172/JCI136098 33720050 PMC7954595

[ref107] Oughtred R. Rust J. Chang C. Breitkreutz B. J. Stark C. Willems A.. (2021). The BioGRID database: a comprehensive biomedical resource of curated protein, genetic, and chemical interactions. Protein Sci. 30, 187–200. doi: 10.1002/pro.3978 33070389 PMC7737760

[ref108] Pagano A. Breuzard G. Parat F. Tchoghandjian A. Figarella-Branger D. De Bessa T. C.. (2021). Tau regulates glioblastoma progression, 3D cell organization, growth and migration via the PI3K-AKT Axis. Cancers 13:5818. doi: 10.3390/cancers13225818 34830972 PMC8616151

[ref109] Pang L. Hu J. Li F. Yuan H. Yan M. Liao G.. (2019). Discovering rare genes contributing to cancer stemness and invasive potential by GBM single-cell transcriptional analysis. Cancers 11:2025. doi: 10.3390/cancers11122025 31888172 PMC6966673

[ref110] Parker N. R. Khong P. Parkinson J. F. Howell V. M. Wheeler H. R. (2015). Molecular heterogeneity in glioblastoma: potential clinical implications. Front. Oncol. 5:55. doi: 10.3389/fonc.2015.00055 25785247 PMC4347445

[ref111] Peignan L. Garrido W. Segura R. Melo R. Rojas D. Carcamo J. G.. (2011). Combined use of anticancer drugs and an inhibitor of multiple drug resistance-associated protein-1 increases sensitivity and decreases survival of glioblastoma multiforme cells in vitro. Neurochem. Res. 36, 1397–1406. doi: 10.1007/s11064-011-0464-8 21544552

[ref112] Perea J. R. Llorens-Martin M. Avila J. Bolos M. (2018). The role of microglia in the spread of tau: relevance for tauopathies. Front. Cell. Neurosci. 12:172. doi: 10.3389/fncel.2018.00172 30042659 PMC6048186

[ref113] Pernia O. Sastre-Perona A. Rodriguez-Antolin C. Garcia-Guede A. Palomares-Bralo M. Rosas R.. (2020). A novel role for the tumor suppressor gene ITF2 in tumorigenesis and chemotherapy response. Cancers 12:786. doi: 10.3390/cancers12040786 32224864 PMC7226299

[ref114] Phoa A. F. Recasens A. Gurgis F. M. S. Betts T. A. Menezes S. V. Chau D.. (2020). MK2 inhibition induces p53-dependent senescence in glioblastoma cells. Cancers 12:654. doi: 10.3390/cancers12030654 32168910 PMC7139970

[ref115] Pillich R. T. Chen J. Churas C. Fong D. Gyori B. M. Ideker T.. (2023). NDEx IQuery: a multi-method network gene set analysis leveraging the network data exchange. Bioinformatics 39:btad118. doi: 10.1093/bioinformatics/btad118, PMID: 36882166 PMC10023220

[ref116] Pokorna M. Hudec M. Jurickova I. Vacha M. Polivkova Z. Kutna V.. (2021). All-trans retinoic acid fosters the multifarious U87MG cell line as a model of glioblastoma. Brain Sci. 11:812. doi: 10.3390/brainsci11060812 34207434 PMC8234004

[ref117] Pollard S. M. Yoshikawa K. Clarke I. D. Danovi D. Stricker S. Russell R.. (2009). Glioma stem cell lines expanded in adherent culture have tumor-specific phenotypes and are suitable for chemical and genetic screens. Cell Stem Cell 4, 568–580. doi: 10.1016/j.stem.2009.03.014 19497285

[ref118] Ponten J. Macintyre E. H. (1968). Long term culture of normal and neoplastic human glia. Acta Pathol. Microbiol. Scand. 74, 465–486. doi: 10.1111/j.1699-0463.1968.tb03502.x 4313504

[ref119] Prabhakar S. Asuthkar S. Lee W. Chigurupati S. Zakharian E. Tsung A. J.. (2014). Targeting DUSPs in glioblastomas - wielding a double-edged sword? Cell Biol. Int. 38, 145–153. doi: 10.1002/cbin.10201 24155099

[ref120] Purow B. (2022). Delivering glioblastoma a kick-DGKalpha inhibition as a promising therapeutic strategy for GBM. Cancers 14:1269. doi: 10.3390/cancers14051269 35267577 PMC8909282

[ref121] Qiu W. Xiao Z. Yang Y. Jiang L. Song S. Qi X.. (2023). USP10 deubiquitinates RUNX1 and promotes proneural-to-mesenchymal transition in glioblastoma. Cell Death Dis. 14:207. doi: 10.1038/s41419-023-05734-y 36949071 PMC10033651

[ref122] Qu H. Jiang W. Wang Y. Chen P. (2019). STOML2 as a novel prognostic biomarker modulates cell proliferation, motility and chemo-sensitivity via IL6-Stat3 pathway in head and neck squamous cell carcinoma. Am. J. Transl. Res. 11, 683–695. 30899371 PMC6413287

[ref123] Qu X. Wu S. Gao J. Qin Z. Zhou Z. Liu J. (2021). Weighted gene co expression network analysis (WGCNA) with key pathways and hub-genes related to micro RNAs in ischemic stroke. IET Syst. Biol. 15, 93–100. doi: 10.1049/syb2.12016 33880887 PMC8675812

[ref124] Rajendran S. Hu Y. Canella A. Peterson C. Gross A. Cam M.. (2023). Single-cell RNA sequencing reveals immunosuppressive myeloid cell diversity during malignant progression in a murine model of glioma. Cell Rep. 42:112197. doi: 10.1016/j.celrep.2023.112197 36871221

[ref125] Ravi V. M. Neidert N. Will P. Joseph K. Maier J. P. Kuckelhaus J.. (2022). T-cell dysfunction in the glioblastoma microenvironment is mediated by myeloid cells releasing interleukin-10. Nat. Commun. 13:925. doi: 10.1038/s41467-022-28523-1 35177622 PMC8854421

[ref126] Riemenschneider M. J. Hegi M. E. Reifenberger G. (2010). MGMT promoter methylation in malignant gliomas. Target. Oncol. 5, 161–165. doi: 10.1007/s11523-010-0153-6 20725792

[ref127] Ritchie M. E. Phipson B. Wu D. Hu Y. Law C. W. Shi W.. (2015). Limma powers differential expression analyses for RNA-sequencing and microarray studies. Nucleic Acids Res. 43:e47. doi: 10.1093/nar/gkv007 25605792 PMC4402510

[ref128] Robin X. Turck N. Hainard A. Tiberti N. Lisacek F. Sanchez J. C.. (2011). pROC: an open-source package for R and S+ to analyze and compare ROC curves. BMC Bioinform. 12:77. doi: 10.1186/1471-2105-12-77 PMC3068975 21414208

[ref129] Rominiyi O. Collis S. J. (2022). DDRugging glioblastoma: understanding and targeting the DNA damage response to improve future therapies. Mol. Oncol. 16, 11–41. doi: 10.1002/1878-0261.13020 34036721 PMC8732357

[ref130] Salvadores M. Fuster-Tormo F. Supek F. (2020). Matching cell lines with cancer type and subtype of origin via mutational, epigenomic, and transcriptomic patterns. Science. Advances 6:eaba1862. doi: 10.1126/sciadv.aba1862 PMC7458440 32937430

[ref131] Salzillo T. C. Hu J. Nguyen L. Whiting N. Lee J. Weygand J.. (2016). Interrogating metabolism in brain cancer. Magn. Reson. Imaging Clin. N. Am. 24, 687–703. doi: 10.1016/j.mric.2016.07.003 27742110 PMC5091807

[ref132] Schaffenrath J. Wyss T. He L. Rushing E. J. Delorenzi M. Vasella F.. (2021). Blood-brain barrier alterations in human brain tumors revealed by genome-wide transcriptomic profiling. Neuro-Oncology 23, 2095–2106. doi: 10.1093/neuonc/noab022 33560373 PMC8643445

[ref133] Shannon P. Markiel A. Ozier O. Baliga N. S. Wang J. T. Ramage D.. (2003). Cytoscape: a software environment for integrated models of biomolecular interaction networks. Genome Res. 13, 2498–2504. doi: 10.1101/gr.1239303 14597658 PMC403769

[ref134] Signorini L. F. Almozlino T. Sharan R. (2021). ANAT 3.0: a framework for elucidating functional protein subnetworks using graph-theoretic and machine learning approaches. BMC Bioinform. 22:526. doi: 10.1186/s12859-021-04449-1 PMC8555137 34706638

[ref135] Singh S. K. Hawkins C. Clarke I. D. Squire J. A. Bayani J. Hide T.. (2004). Identification of human brain tumour initiating cells. Nature 432, 396–401. doi: 10.1038/nature03128 15549107

[ref136] Sirvinskas D. Steponaitis G. Stakaitis R. Tamasauskas A. Vaitkiene P. Skiriute D. (2023). Antisense lncRNA CHROMR is linked to glioma patient survival. Front. Mol. Biosci. 10:1101953. doi: 10.3389/fmolb.2023.1101953 36950523 PMC10025505

[ref137] Smith A. L. Gjoka E. Izhar M. Novo K. J. Mason B. C. De Las Casas A.. (2021). FGGY carbohydrate kinase domain containing is expressed and alternatively spliced in skeletal muscle and attenuates MAP kinase and Akt signaling. Gene 800:145836. doi: 10.1016/j.gene.2021.145836 34280510

[ref138] Song X. Zhou K. Zhao Y. Huai C. Zhao Y. Yu H.. (2012). Fine mapping analysis of a region of 20q13.33 identified five independent susceptibility loci for glioma in a Chinese Han population. Carcinogenesis 33, 1065–1071. doi: 10.1093/carcin/bgs117 22387365

[ref139] Srivastava S. Jackson C. Kim T. Choi J. Lim M. (2019). A characterization of dendritic cells and their role in immunotherapy in glioblastoma: from preclinical studies to clinical trials. Cancers 11:537. doi: 10.3390/cancers11040537 30991681 PMC6521200

[ref140] Stupp R. Hegi M. E. Mason W. P. van den Bent M. J. Taphoorn M. J. Janzer R. C.. (2009). Effects of radiotherapy with concomitant and adjuvant temozolomide versus radiotherapy alone on survival in glioblastoma in a randomised phase III study: 5-year analysis of the EORTC-NCIC trial. Lancet Oncol. 10, 459–466. doi: 10.1016/S1470-2045(09)70025-7 19269895

[ref141] Sun P. Xia S. Lal B. Shi X. Yang K. S. Watkins P. A.. (2014). Lipid metabolism enzyme ACSVL3 supports glioblastoma stem cell maintenance and tumorigenicity. BMC Cancer 14:401. doi: 10.1186/1471-2407-14-401 24893952 PMC4055398

[ref142] Sun X. Zhang J. Xiao C. Ge Z. (2022). Expression profile and prognostic values of LSM family in skin cutaneous melanoma. BMC Med. Genet. 15:238. doi: 10.1186/s12920-022-01395-6 PMC9656080 36371223

[ref143] Tang M. Bolderson E. O'Byrne K. J. Richard D. J. (2021). Tumor hypoxia drives genomic instability. Front. Cell Dev. Biol. 9:626229. doi: 10.3389/fcell.2021.626229 33796526 PMC8007910

[ref144] Taouis K. Driouch K. Lidereau R. Lallemand F. (2021). Molecular functions of WWOX potentially involved in cancer development. Cells 10:1051. doi: 10.3390/cells10051051 33946771 PMC8145924

[ref145] Thwaites D. T. Anderson C. M. (2011). The SLC36 family of proton-coupled amino acid transporters and their potential role in drug transport. Br. J. Pharmacol. 164, 1802–1816. doi: 10.1111/j.1476-5381.2011.01438.x 21501141 PMC3246705

[ref146] Tomi-Andrino C. Pandele A. Winzer K. King J. Rahman R. Kim D. H. (2022). Metabolic modeling-based drug repurposing in glioblastoma. Sci. Rep. 12:11189. doi: 10.1038/s41598-022-14721-w 35778411 PMC9249780

[ref147] Varricchio A. Khan S. Price Z. K. Davis R. A. Ramesh S. A. Yool A. J. (2023). Pharmacological inhibition of membrane signaling mechanisms reduces the invasiveness of U87-MG and U251-MG glioblastoma cells *in vitro*. Cancers 15:1027. doi: 10.3390/cancers15041027 36831372 PMC9954756

[ref148] Waha A. Felsberg J. Hartmann W. von dem Knesebeck A. Mikeska T. Joos S.. (2010). Epigenetic downregulation of mitogen-activated protein kinase phosphatase MKP-2 relieves its growth suppressive activity in glioma cells. Cancer Res. 70, 1689–1699. doi: 10.1158/0008-5472.CAN-09-3218 20124482

[ref149] Wan X. Guan S. Hou Y. Qin Y. Zeng H. Yang L.. (2021). FOSL2 promotes VEGF-independent angiogenesis by transcriptionnally activating Wnt5a in breast cancer-associated fibroblasts. Theranostics 11, 4975–4991. doi: 10.7150/thno.55074 33754039 PMC7978317

[ref150] Wang F. Peng L. Sun Y. Zhang B. Lu S. (2022). PUF60 promotes glioblastoma progression through regulation of EGFR stability. Biochem. Biophys. Res. Commun. 636, 190–196. doi: 10.1016/j.bbrc.2022.10.082 36335869

[ref151] Wang F. Yao F. Zengyan L. (2021). Effect of miR-7-5p on proliferation, invasion of non-small cell lung cancer cells by targeting POLE4 and its underlying mechanism. Cancer Res. Prev. Treat. 48, 709–713. doi: 10.3971/j.issn.1000-8578.2021.20.1275

[ref152] Wang H. Y. Li J. Y. Liu X. Yan X. Y. Wang W. Wu F.. (2016). A three ion channel genes-based signature predicts prognosis of primary glioblastoma patients and reveals a chemotherapy sensitive subtype. Oncotarget 7, 74895–74903. doi: 10.18632/oncotarget.12462 27713134 PMC5342710

[ref153] Wang L. B. Karpova A. Gritsenko M. A. Kyle J. E. Cao S. Li Y.. (2021). Proteogenomic and metabolomic characterization of human glioblastoma. Cancer Cell 39, 509–528 e520. doi: 10.1016/j.ccell.2021.01.006 33577785 PMC8044053

[ref154] Wang Q. Hu B. Hu X. Kim H. Squatrito M. Scarpace L.. (2017). Tumor evolution of glioma-intrinsic gene expression subtypes associates with immunological changes in the microenvironment. Cancer Cell 32, 42–56 e46. doi: 10.1016/j.ccell.2017.06.003 28697342 PMC5599156

[ref155] Wei W. Xu R. Ying X. Chen L. Lu X. Tang Q.. (2022). Transcriptome analysis of solute carrier-associated genes in hepatocellular carcinoma: friend or foe? Front. Genet. 13:856393. doi: 10.3389/fgene.2022.856393 35401672 PMC8984160

[ref156] Winardi W. Tsai C. Y. Chen W. T. Tsai H. P. Chung C. L. Loh J. K.. (2013). Reduced WWOX protein expression in human astrocytoma. Neuropathology 33, 621–627. doi: 10.1111/neup.12040 23675860

[ref157] Wiszniak S. Mackenzie F. E. Anderson P. Kabbara S. Ruhrberg C. Schwarz Q. (2015). Neural crest cell-derived VEGF promotes embryonic jaw extension. Proc. Natl. Acad. Sci. U. S. A. 112, 6086–6091. doi: 10.1073/pnas.1419368112 25922531 PMC4434710

[ref158] Yaghi L. Poras I. Simoes R. T. Donadi E. A. Tost J. Daunay A.. (2016). Hypoxia inducible factor-1 mediates the expression of the immune checkpoint HLA-G in glioma cells through hypoxia response element located in exon 2. Oncotarget 7, 63690–63707. doi: 10.18632/oncotarget.11628 27577073 PMC5325396

[ref159] Yi S.-J. Jang Y.-J. Lee S. Cho S.-J. Kang K. Park J.-I.. (2023). TMBIM6 deficiency leads to bone loss by accelerating osteoclastogenesis. Redox Biol. 64:102804. doi: 10.1016/j.redox.2023.102804 37399733 PMC10336580

[ref160] Zakharova G. Efimov V. Raevskiy M. Rumiantsev P. Gudkov A. Belogurova-Ovchinnikova O.. (2022). Reclassification of TCGA diffuse glioma profiles linked to transcriptomic, epigenetic, genomic and clinical data, according to the 2021 WHO CNS tumor classification. Int. J. Mol. Sci. 24:157. doi: 10.3390/ijms24010157 36613601 PMC9820617

[ref161] Zheng X. J. Chen W. L. Yi J. Li W. Liu J. Y. Fu W. Q.. (2022). Apolipoprotein C1 promotes glioblastoma tumorigenesis by reducing KEAP1/NRF2 and CBS-regulated ferroptosis. Acta Pharmacol. Sin. 43, 2977–2992. doi: 10.1038/s41401-022-00917-3 35581292 PMC9622891

[ref162] Zhong J. Bach C. T. Shum M. S. Y. O'Neill G. M. (2014). NEDD9 regulates 3D migratory activity independent of the Rac1 morphology switch in glioma and neuroblastoma. Mol. Cancer Res. 12, 264–273. doi: 10.1158/1541-7786.Mcr-13-0513 24337070

[ref163] Zhou J. Li L. Jia M. Liao Q. Peng G. Luo G.. (2023). Dendritic cell vaccines improve the glioma microenvironment: influence, challenges, and future directions. Cancer Med. 12, 7207–7221. doi: 10.1002/cam4.5511 36464889 PMC10067114

[ref164] Zhu G. D. Yu J. Sun Z. Y. Chen Y. Zheng H. M. Lin M. L.. (2021). Genome-wide CRISPR/Cas9 screening identifies CARHSP1 responsible for radiation resistance in glioblastoma. Cell Death Dis. 12:724. doi: 10.1038/s41419-021-04000-3 34290231 PMC8295287

