# Supplementary figures and images for: Molecular landscapes of glioblastoma cell lines revealed a group of patients that do not benefit from WWOX tumor suppressor expression
Source: Front Neurosci. 2023 Sep 15;17:1260409. doi: 10.3389/fnins.2023.1260409 (PMC10540236; doi:10.3389/fnins.2023.1260409)

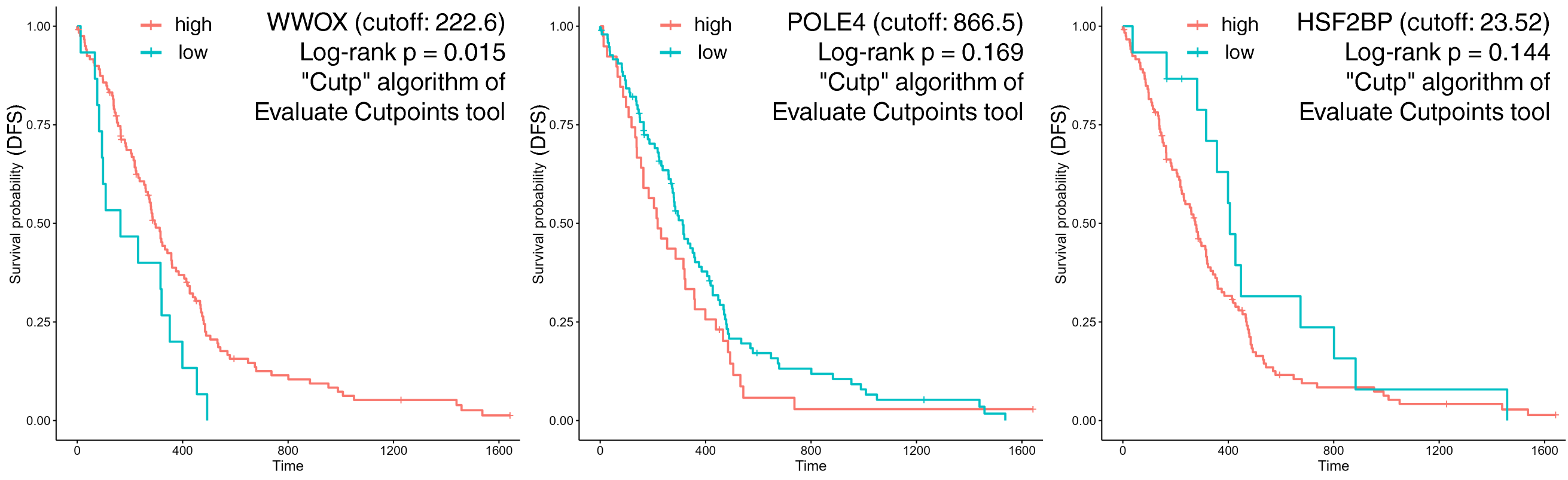

Supplement: Supplementary file 1 [file Image_1.TIF]

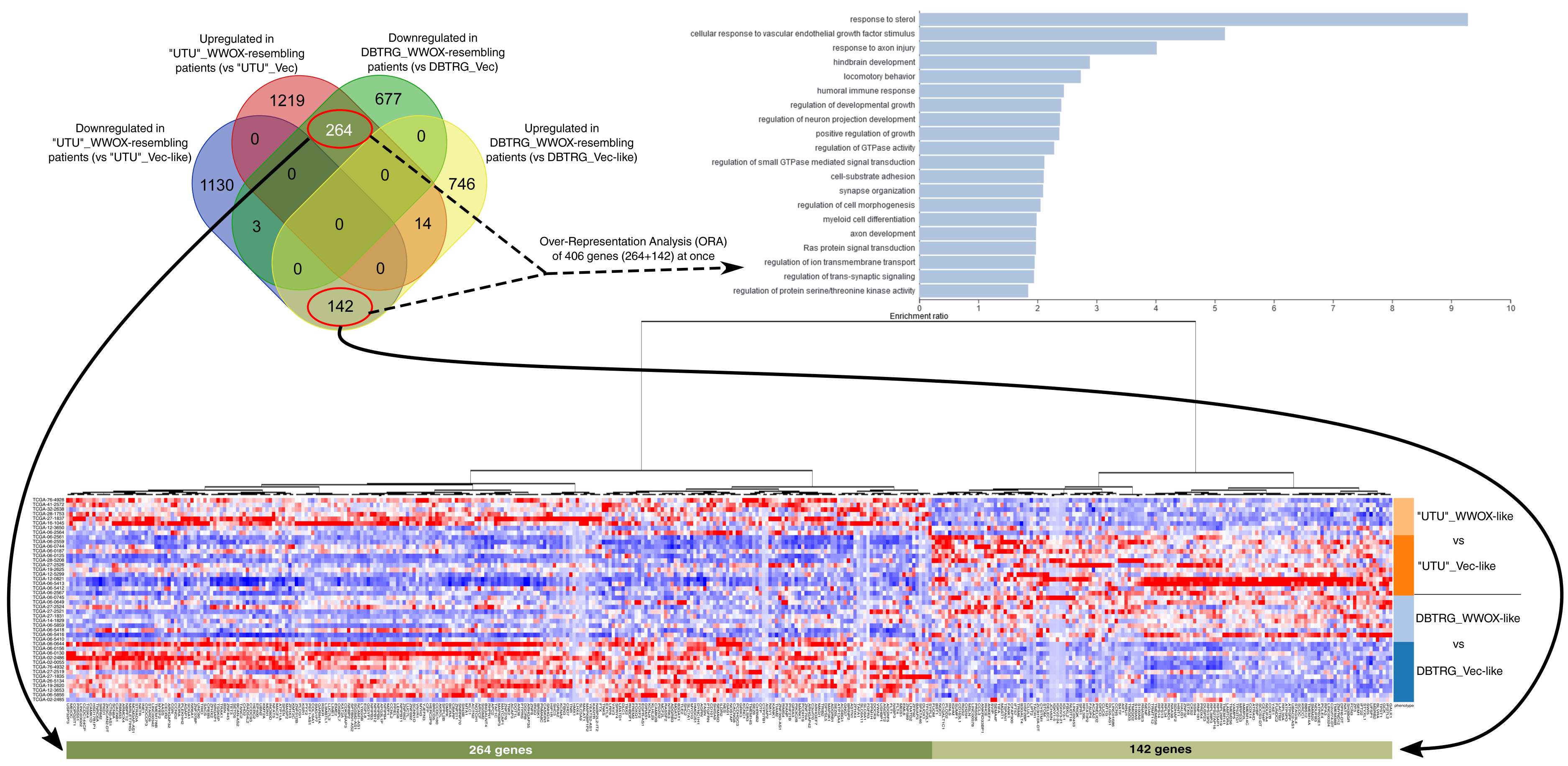

Supplement: Supplementary file 2 [file Image_2.TIF]

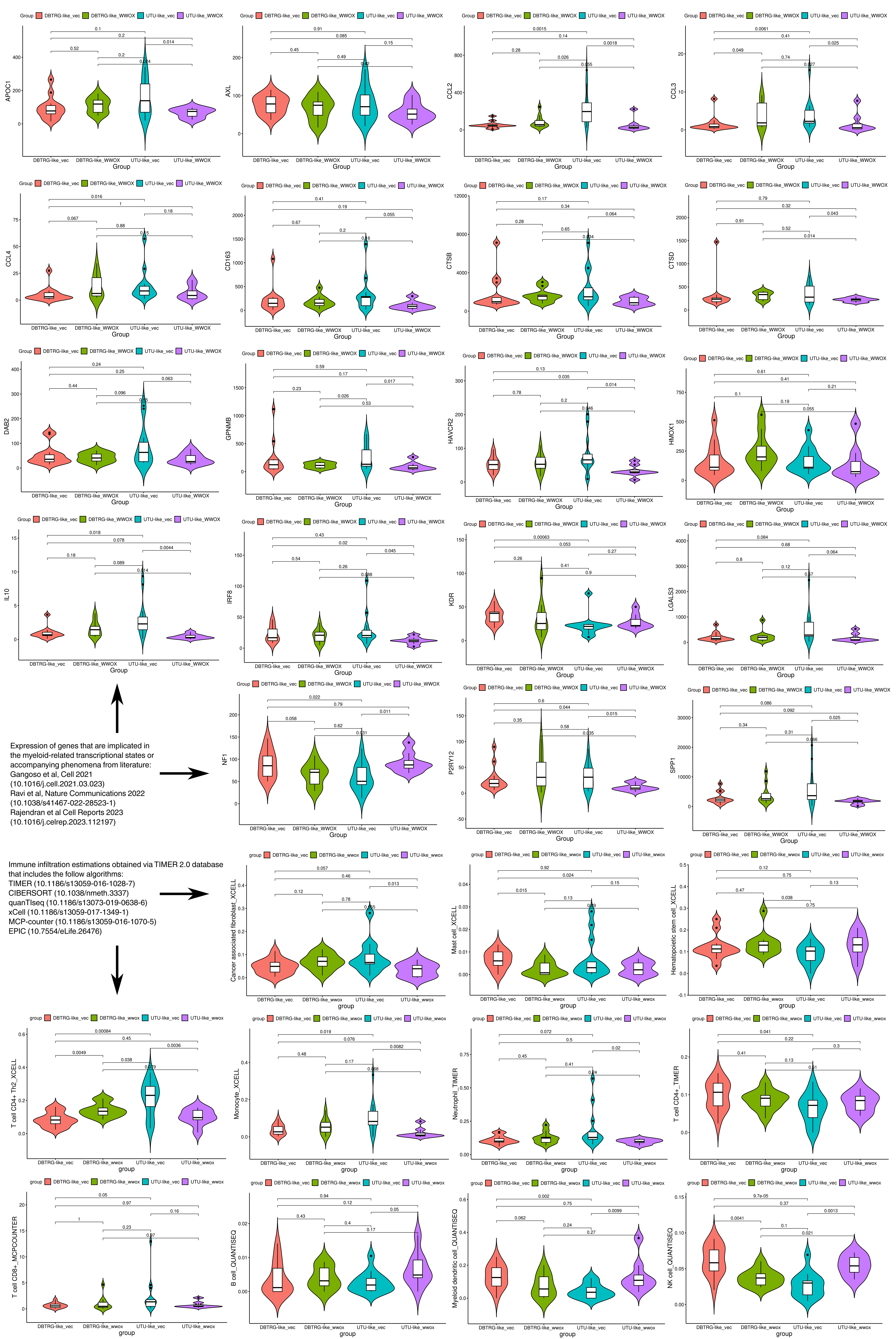

Supplement: Supplementary file 3 [file Image_3.TIF]

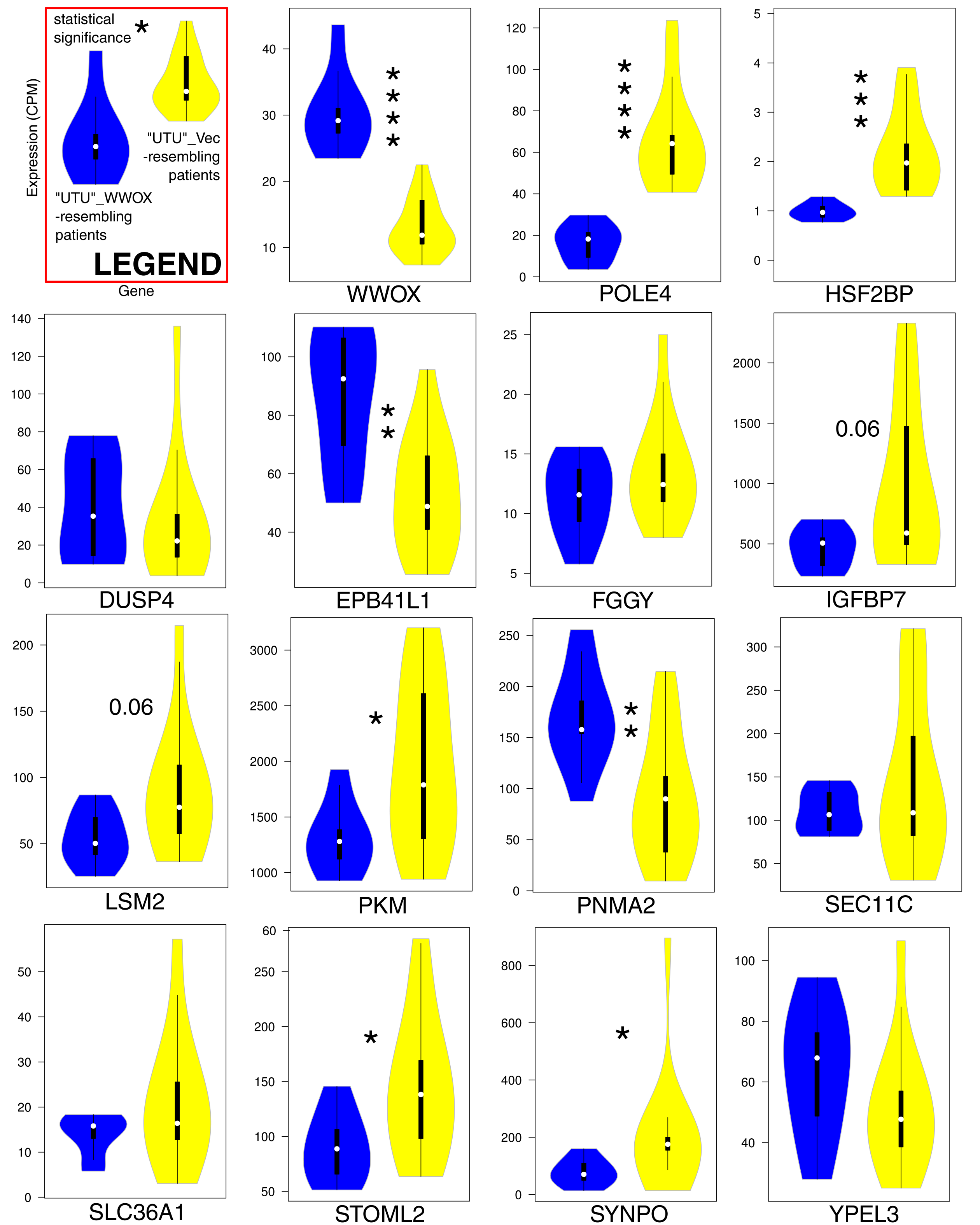

Supplement: Supplementary file 4 [file Image_4.TIF]

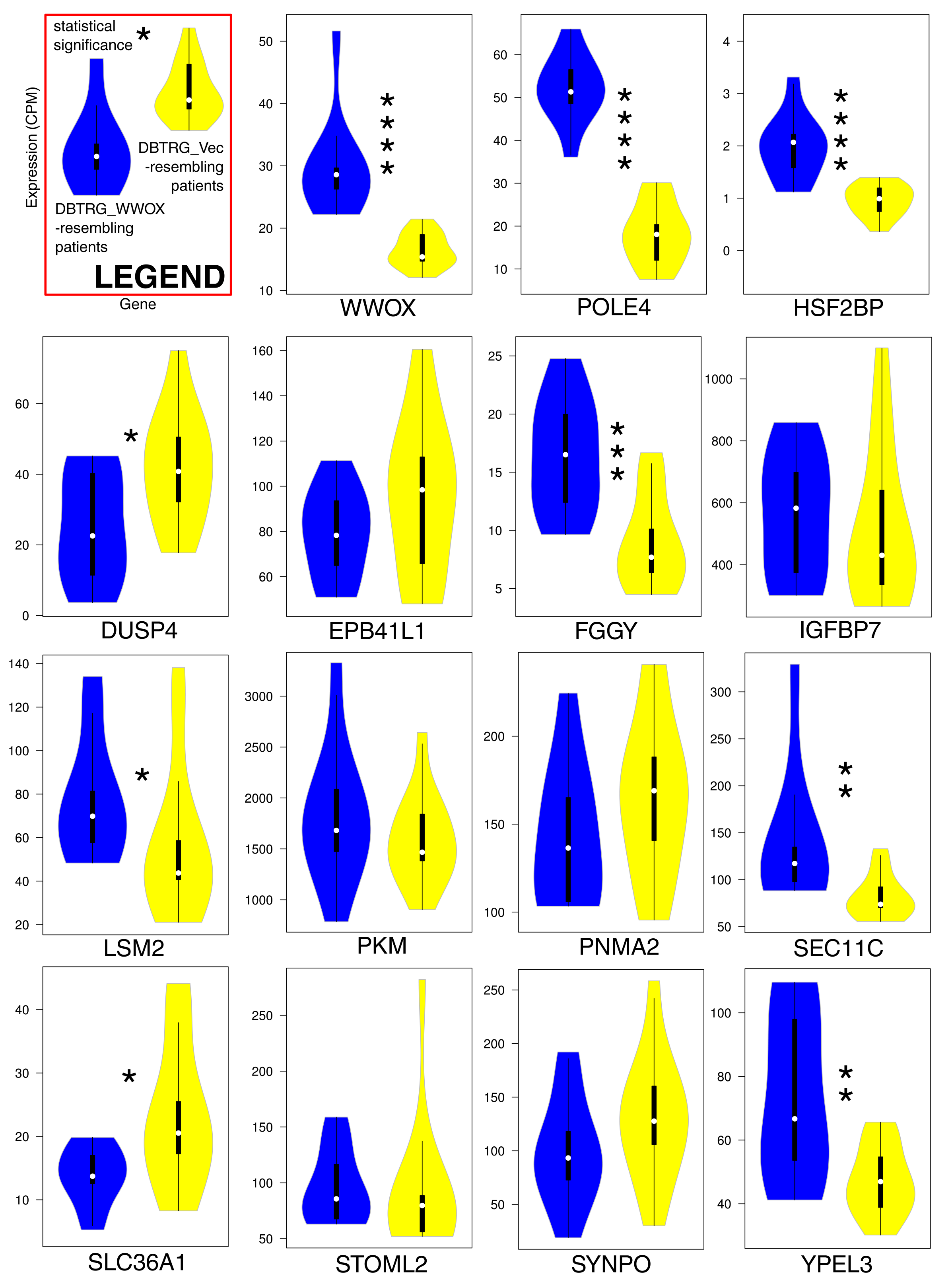

Supplement: Supplementary file 5 [file Image_5.TIF]
